# Role of AMPK-SREBP Signaling in Regulating Fatty Acid Binding-4 (FABP4) Expression following Ethanol Metabolism

**DOI:** 10.3390/biology11111613

**Published:** 2022-11-04

**Authors:** Neha Attal, Emilio Marrero, Kyle J. Thompson, Iain H. McKillop

**Affiliations:** Department of Surgery, Atrium Health-Carolinas Medical Center, 1000 Blythe Blvd., Charlotte, NC 28203, USA

**Keywords:** alcohol, liver disease, cytochrome P4502E1, SREBP-1c, AMPKα, fatty acid binding protein

## Abstract

**Simple Summary:**

Liver damage is a common occurrence following sustained, heavy alcohol consumption. One of the earliest pathologies associated with alcohol-dependent liver disease is increased hepatic fat accumulation. In healthy individuals, fatty acid binding protein-1 plays an important role in transporting lipids in hepatocytes. However, in alcohol-dependent liver disease, fatty acid binding protein-4 (a protein normally expressed in fat storing cells and white blood cells) is produced by hepatocytes and can stimulate liver cancer growth. In this study, we report that alcohol metabolism via cytochrome P450 2E1 drives increased fatty acid binding protein-4 production and fat accumulation. These data suggest that increased fatty acid binding protein-4 production may promote tumor growth in cancer cells in alcohol-dependent liver disease.

**Abstract:**

Fatty acid binding protein-4 (*FABP4*) is not normally expressed in the liver but is induced in alcohol-dependent liver disease (ALD)). This study sought to identify mechanisms whereby ethanol (EtOH) metabolism alters triglyceride accumulation and *FABP4* production. Human hepatoma cells which were stably transfected to express alcohol dehydrogenase (ADH) or cytochrome P4502E1 (CYP2E1) were exposed to EtOH in the absence/presence of inhibitors of ADH (4-methylpyrazole) or CYP2E1 (chlormethiazole). Cells were analyzed for free fatty acid (FFA) content and *FABP4* mRNA, then culture medium assayed for *FABP4* levels. Cell lysates were analyzed for AMP-activated protein kinase-α (AMPKα), Acetyl-CoA carboxylase (ACC), sterol regulatory element binding protein-1c (SREBP-1c), and Lipin-1β activity and localization in the absence/presence of EtOH and pharmacological inhibitors. CYP2E1-EtOH metabolism led to increased *FABP4* mRNA/protein expression and FFA accumulation. Analysis of signaling pathway activity revealed decreased AMPKα activation and increased nuclear-SREBP-1c localization following CYP2E1-EtOH metabolism. The role of AMPKα-SREBP-1c in regulating CYP2E1-EtOH-dependent FFA accumulation and increased *FABP4* was confirmed using pharmacological inhibitors and over-expression of AMPKα. Inhibition of ACC or Lipin-1β failed to prevent FFA accumulation or changes in *FABP4* mRNA expression or protein secretion. These data suggest that CYP2E1-EtOH metabolism inhibits AMPKα phosphorylation to stimulate FFA accumulation and *FABP4* protein secretion via an SREBP-1c dependent mechanism.

## 1. Introduction

Alcohol-dependent liver disease (ALD) manifests as progressively worsening liver health following heavy ethanol (EtOH) ingestion, usually occurring over a period of years to decades [1]. The early stages of ALD are characterized by increased lipid accumulation in hepatocytes (hepatosteatosis) [1,2]. While hepatosteatosis can be readily detected using invasive (biopsy) or noninvasive (Fibroscan) means, it is rarely diagnosed in patients due to being predominantly asymptomatic. In the absence of abstinence, continued heavy EtOH ingestion often causes ALD progression from hepatosteatosis to alcoholic hepatitis, fibrosis, and cirrhosis [3]. As ALD progresses, a series of intrahepatic and systemic responses to sustained metabolic and chemical insult contrive to increase the risk of genetic damage, underlying hepatic cirrhosis and development of hepatocellular carcinoma (HCC) [1].

Hepatic sequestration and processing of lipids is a critical homeostatic function. In healthy hepatocytes free fatty acid (FFA) sequestration, transport, and storage occurs via a number of integrated pathways which include fatty acid binding protein-1 (FABP1) [4]. More recently, *FABP4* (a member of the FABP family that is normally expressed in adipocytes and macrophages [5]) is reported to be elevated in rodent models of both ALD and non-alcoholic fatty liver disease (NAFLD), as well as in serum and hepatic tissue from patients diagnosed with ALD or NAFLD [6,7]. Furthermore, adipocyte-derived *FABP4* has been identified as a potential endocrine signaling molecule in vasculature [8] and tumors located adjacent to adipose tissue [9]. Exogenous *FABP4* stimulates tumor cell growth and migration in a number of different cancers, including hepatomas [7,9].

Following ingestion and intestinal adsorption, the liver is the major site of EtOH metabolism [1]. In the setting of moderate EtOH consumption, alcohol dehydrogenase (ADH) rapidly oxidizes EtOH to acetaldehyde [10]. However, in the setting of sustained, heavy EtOH intake, cytochrome P450 2E1 (CYP2E1) is induced, and oxidizes EtOH to acetaldehyde [11]. In addition to increased toxicity resulting from elevated hepatic acetaldehyde levels, the metabolism of EtOH by CYP2E1 also leads to increased reactive oxygen species (ROS) production and intracellular oxidative stress, amplifying cellular lipid, protein, and nucleic acid damage [11].

Accumulating evidence indicates that EtOH-mediated fat accumulation in hepatocytes is significantly impacted by inhibition of the sirtunin-1-AMP-activated protein kinase (SIRT1-AMPKα) axis, and that decreased SIRT1-AMPKα signaling alters the activity of a number of downstream signaling pathways to reduce FFA oxidation/promote lipogenesis [12]. Under these circumstances, the net increase in intracellular lipid content may underlie the increased *FABP4* production detected in ALD [6,7]. Previous studies report that CYP2E1-EtOH metabolism inhibits SIRT1 and promotes forkhead Box O1 (FOXO1) nuclear localization to alter transcription factor activity and reduce fatty acid oxidation. This leads to elevated intracellular fatty acid accumulation and increased *FABP4* synthesis/release [7]. The effects of EtOH on lipid accumulation/FABP4 production are abrogated by inhibiting CYP2E1 or exposing cells to pharmacological inhibitors of SIRT1/FOXO1 [7]. Previous studies report that inhibition of SIRT1 by EtOH metabolism prevents SIRT1 mediated activation of AMPKα via upregulation of the upstream AMPK, liver kinase B1 (LKB1) [13]. In addition, increased intracellular ROS/acetaldehyde resulting from EtOH metabolism is reported to inhibit AMPKα phosphorylation by attenuating LKB1, the effect of which is to alter downstream signaling via changes in sterol regulatory element binding protein 1 (SREBP-1c), Acetyl-CoA carboxylase (ACC), and Lipin-1β activity/localization, ultimately resulting in cellular lipid accumulation [13].

This study sought to analyze the role of EtOH-metabolism (ADH and CYP2E1-dependent) on AMPKα activity, and to identify downstream effectors involved in regulating intracellular FFA accumulation and *FABP4* expression.

## 2. Materials and Methods

### 2.1. Cell Culture

The human HepG2 cell line was purchased from the American Type Culture Collection (Manassas, VA, USA) and the human HuH7 cell line was purchased from the Japanese Collection of Research Bioresources Cell Bank (Sekisui XenoTech; Kansas City, KS, USA). HepG2 cells stably transfected to express CYP2E1 (E47 [14]) were provided by Dr. A. Cederbaum (Ichan School of Medicine, New York, NY, USA). HepG2 cells which were stably transfected to express ADH (VA-13^ADH+^ [15]) were provided by Dr. D.L. Clemens (University of Nebraska, Omaha, NE, USA). HuH7 cells which were stably transfected to express CYP2E1 (HuH7^CYP+^ [16]) were provided by Dr. N. Osna (University of Nebraska).

### 2.2. Culture Conditions

Cell culture was performed using high glucose Dulbecco’s Modified Eagle Medium (DMEM; Gibco, Waltham, MA, USA) with 10% (*v*/*v*) fetal bovine serum (FBS) as previously reported [17].

To assess the potential role of acetaldehyde in regulating changes in *FABP4*, VA-13^ADH+^ cells were exposed to EtOH (50 mM). In parallel, HepG2 and HuH7 cells were exposed to an acetaldehyde generating system (AGS) for 48 h [17]. At experiment completion culture medium was collected and cells processed for RNA/protein analysis.

To assess the impact of EtOH metabolism on changes in *FABP4* mRNA and *FABP4* protein levels HepG2 and E47 cells, HuH7 and HuH7^CYP+^ cells, and HepG2/VA-13^ADH+^ cells were maintained in low serum medium (0.1% (*v*/*v*) FBS-DMEM (LSM)) for 24 h prior to EtOH addition (0–100 mM, 48-h). Alternatively, CYP2E1 or ADH expressing cells were exposed to EtOH (50 mM) in the presence of chlormethiazole (CMZ, 100 µM, Sigma-Aldrich, St. Louis, MO, USA) or 4-methylpyrazole (4 MP; 5 mMol, Sigma-Aldrich), respectively. Forty-eight hours later, the culture medium was collected and cells processed for RNA/protein analysis.

### 2.3. Use of Pharmacological Inhibitors of Cell Signaling

Cells were maintained in LSM (24-h) prior to the addition of inhibitors of AMPKα (Compound C; 50 µM [18]), SREBP-1c (Fatostatin; 10 µM [19]), ACC (Cpd9; 20 nM [20]), or Lipin-1/2 (propranolol hydrochloride (PHC); 10 µM [21]) for 1 h, followed by the addition of EtOH (50 mM). Forty-eight hours later, the culture medium was collected, and cells were processed for RNA/protein analysis.

### 2.4. RNA Analysis

Total RNA extraction was performed (Quick-RNA^TM^ Miniprep, Zymo Research, Tustin, CA, USA) and reverse transcribed to cDNA (IMPROM II^TM^, Promega, Madison, WI, USA). Using TaqMan probes for *FABP4* and AMPKα (Thermo Fisher Scientific, Grand Island, NY, USA) quantitative RT-PCR (qRT-PCR) was performed (TaqMan Universal Master Mix II, Thermo Fisher Scientific). *FABP4* and *AMPK*α mRNA expression was calculated and normalized to 18s-RNA [17].

### 2.5. Protein Detection

#### 2.5.1. Enzyme-Linked Immunosorbent Assay (ELISA)

Abundance of *FABP4* protein in culture medium was detected using a commercial enzyme-linked immunosorbent assay (R & D Systems, Minneapolis, MN, USA).

#### 2.5.2. Western Blot Analysis

To determine relative protein abundance, cell lysates were prepared using a radioimmuno-precipitation assay (RIPA) buffer with phosphatase and protease inhibitors. Protein concentrations were equalized, and total/phosphorylated AMPKα (AMPKα/pAMPKα), precursor SREBP-1c/mature SREBP-1c (pre-SREBP-1c/mat-SREBP-1c), total and phosphorylated-ACC (ACC/pACC), and Lipin-1β were detected by Western blot using antibodies against AMPKα/pAMPKα (Thr172) (Cell Signaling Technologies, Danvers, MA, USA), ACC/pACC (Cell Signaling Technologies), SREBP-1c (Santa Cruz Biotechnology, Dallas, TX, USA), or Lipin-1β (Abcam, Waltham, MA, USA).

To assess protein localization, nuclear and cytoplasmic cell fractions were prepared using NE-PER^TM^ nuclear and cytoplasmic extraction reagents (Pierce Biotechnology, Rockford, IL, USA). To confirm the purity of nuclear and cytoplasmic cell fractions, samples were analyzed by Western blot using antibodies against histone 3 (nuclear marker) or glyceraldehye-3-phosphate dehydrogenase (GAPDH; cytoplasmic marker) prior to Western blot analysis of nuclear SREBP-1c and cytoplasmic Lipin-1β, respectively.

Following image capture, densitometric analysis was performed (ImageJ, National Institutes of Health, Bethesda, MD, USA) and protein loading correction was performed to Ponceau S membrane staining or the ratio of phosphorylated to total protein, as appropriate [17].

#### 2.5.3. AMPKα Over-Expression

Using a pCMV6-AC-GFP plasmid constructed to express the *AMPKα Gene* (NM_006251 (Catalog # RG218572); Origene Technologies, Rockville, MD, USA) and green fluorescent protein (GFP), HepG2/E47 or HuH7/HuH7^CYP+^ cells were transfected using Lipofectamine 3000 (Thermo Fisher Scientific). Transfection efficiency was established microscopically and by Western blot using antibodies against AMPKα or GFP (Sigma-Aldrich). Transfected cells were then exposed to EtOH (50 mM, 48-h), culture medium was collected, and cells were processed for RNA/protein analysis.

#### 2.5.4. Change in Cellular Free Fatty Acid (FFA)

Changes in intracellular FFA levels were determined using an FFA Assay kit (Abcam). Briefly, cells were homogenized in a chloroform/Triton-X-100 solution and centrifuged (16,000× *g*, 5 min). The organic phase was collected, air dried (50 °C, 30 min), and vacuum dried (30 min) prior to dissolving in the fatty acid assay buffer solution and the addition of acetyl-coA synthase reagent. A 50 µL aliquot was removed, and absorbance was recorded on a microplate reader at (570 nm).

#### 2.5.5. Quantification and Statistical Analysis

All experiments were performed a minimum of three times. Data are expressed as mean ± SEM and analyzed using a one- or two-way ANOVA, as appropriate (R statistical software (V.3.5.3)). A *p*-value < 0.05 was considered statistically significant.

## 3. Results

### 3.1. CYP2E1 but Not ADH EtOH Metabolism Alters *FABP4* and Intracellular FFA Levels

**Figure 1:**
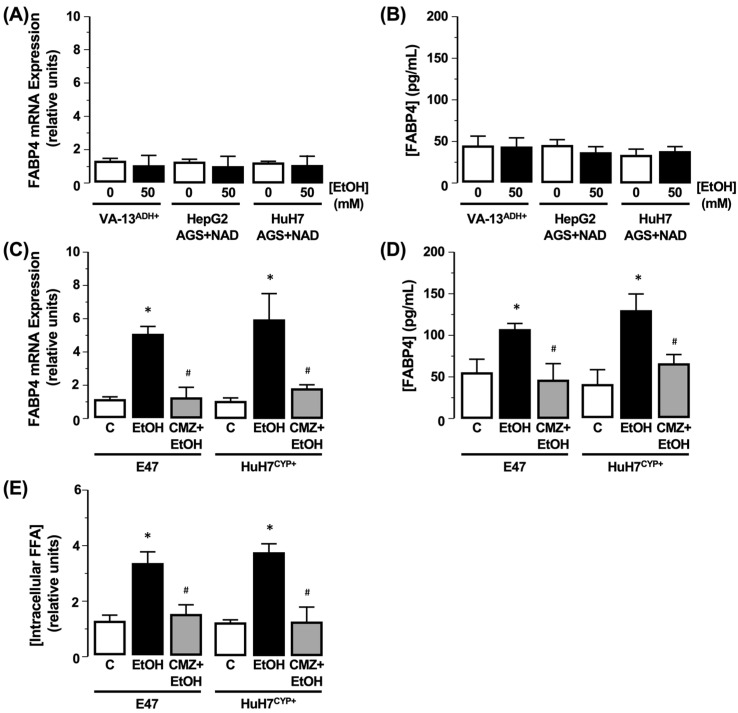
Role of ethanol (EtOH) metabolism on *FABP4* expression and FFA accumulation. HepG2 cells stably transfected to express alcohol dehydrogenase (VA-13^ADH+^) were exposed to EtOH (50 mM), or HepG2/HuH7 cells were exposed to an acetaldehyde generating system (AGS), and (**A**) *FABP4* mRNA and (**B**) *FABP4* protein in the culture medium were measured. N = 3. HepG2 and HuH7 cells transfected to express CYP2E1 (E47 and HuH7^CYP+^) in the absence or presence of chlormethiazole (CMZ; 100 µM) were exposed to EtOH (50 mM) and (**C**) *FABP4* mRNA expression, (**D**) *FABP4* protein in culture medium, and (**E**) cellular FFA was measured. C = 0 mM EtOH. N = 3, * *p* < 0.05 EtOH vs. C, ^#^ *p* < 0.05 CMZ + EtOH vs. EtOH.

HepG2 cells transfected to express ADH (VA-13^ADH+^) demonstrated no change in *FABP4* mRNA expression or *FABP4* protein in culture medium following exposure to EtOH (50 mM), compared to the control (0 mM EtOH), and effects were mirrored in HepG2 or HuH7 cells exposed to an AGS (Figure 1A,B, N = 3). This remained the case in the presence of the ADH inhibitor 4-MP (data not shown). Conversely, cells transfected to express CYP2E1 (E47 and HuH7^CYP+^) demonstrated increased *FABP4* mRNA expression and *FABP4* detected in the culture medium (compared to 0 mM EtOH). This was concomitant with elevated intracellular FFA accumulation, an effect that was inhibited by CMZ (Figure 1C–E, N = 3, * *p* < 0.05 EtOH vs. control, ^#^ *p* < 0.05 CMZ + 50 mM EtOH vs. 50 mM EtOH).

### 3.2. Role of AMPKα in Regulating FABP4 Expression and Fatty Acid Accumulation

**Figure 2:**
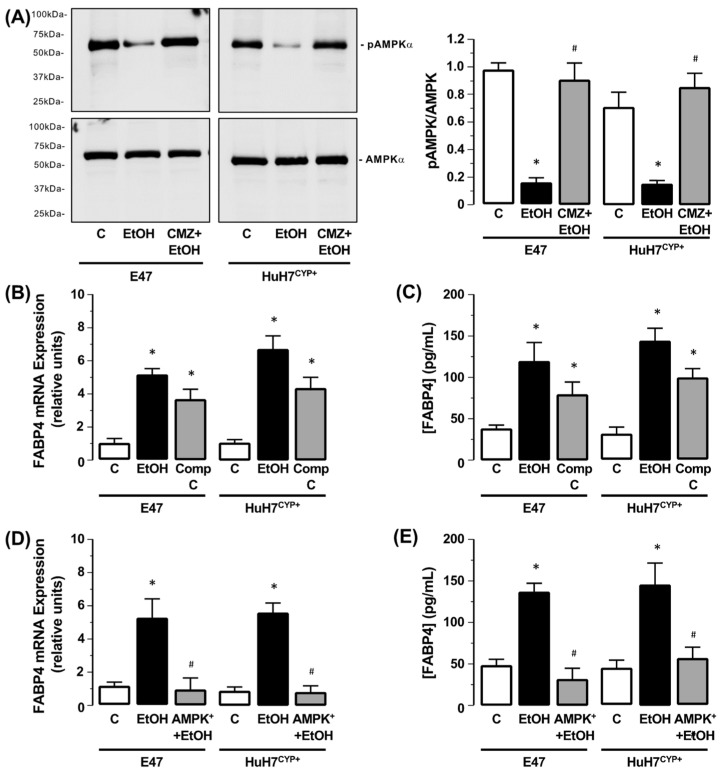
CYP2E1-EtOH metabolism inhibits AMPKα activity. (**A**) Representative Western blot of phospho-AMPKα (pAMPKα) and total AMPKα in CYP2E1-expressing HepG2 (E47) and HuH7 (HuH7^CYP+^) cells after EtOH exposure (50 mM), in the absence or presence of chlormethiazole (CMZ). Protein detection is expressed as ratio of pAMPKα:AMPKα. C = 0 mM EtOH. N = 3, * *p* < 0.05 EtOH vs. C, ^#^ *p* < 0.05 CMZ + EtOH vs. EtOH. Effect of EtOH (50 mM) on E47 and HuH7^CYP+^ cells in the absence or presence of Compound C (Comp C; 50 µM) on (**B**) *FABP4* mRNA and (**C**) *FABP4* protein in the culture medium. N = 3, * *p* < 0.05 EtOH vs. C, ^#^ *p* < 0.05 Comp C vs. EtOH. Effect of EtOH (50 mM) on E47/HuH7^CYP+^ cells and E47/HuH7^CYP+^ cells transfected to overexpress AMPKα (AMPK^+^) on (**D**) *FABP4* mRNA and (**E**) *FABP4* protein in the culture medium. N = 3, * *p* < 0.05 EtOH vs. C, ^#^ *p* < 0.05 AMPK^+^ + EtOH vs. 50 mM EtOH.

No change in AMPK*α*/pAMPK*α* protein expression was detected in HepG2 or HuH7 cells following EtOH exposure compared to the control (0 mM EtOH) (data not shown). Conversely, exposure of E47 and HuH7^CYP+^ cells to EtOH inhibited the amount of pAMPK*α* detected compared to the control (0 mM EtOH). In the absence of changes in total AMPK*α*, effects were abolished in the presence of CMZ (Figure 2A and Figure S1).

Inhibition of AMPK*α* (Compound C, 50 µM) led to increased *FABP4* mRNA expression and *FABP4* protein in the culture medium (Figure 2B,C, N = 3, * *p* < 0.05 vs. control (0 mM EtOH)). Conversely, E47 and HuH7^CYP+^ cells transfected to overexpress *AMPKα* (and *GFP)* (Supplemental Figure S2) no longer exhibited increased *FABP4* expression following EtOH exposure (50 mM) (Figure 2D,E, N = 3, * *p* < 0.05 EtOH vs. C, ^#^ *p* < 0.05 EtOH vs. AMPK^+^ + EtOH). In HepG2 and HuH7 cells, EtOH failed to alter *FABP4* expression, and this remained the case in cells transfected to express AMPK^+^ following EtOH exposure (50 mM) (data not shown).

### 3.3. Role of Downstream AMPKα Effectors in Regulating FABP4 and Cellular Steatosis

#### 3.3.1. Acetyl-CoA Carboxylase (ACC)

**Figure 3:**
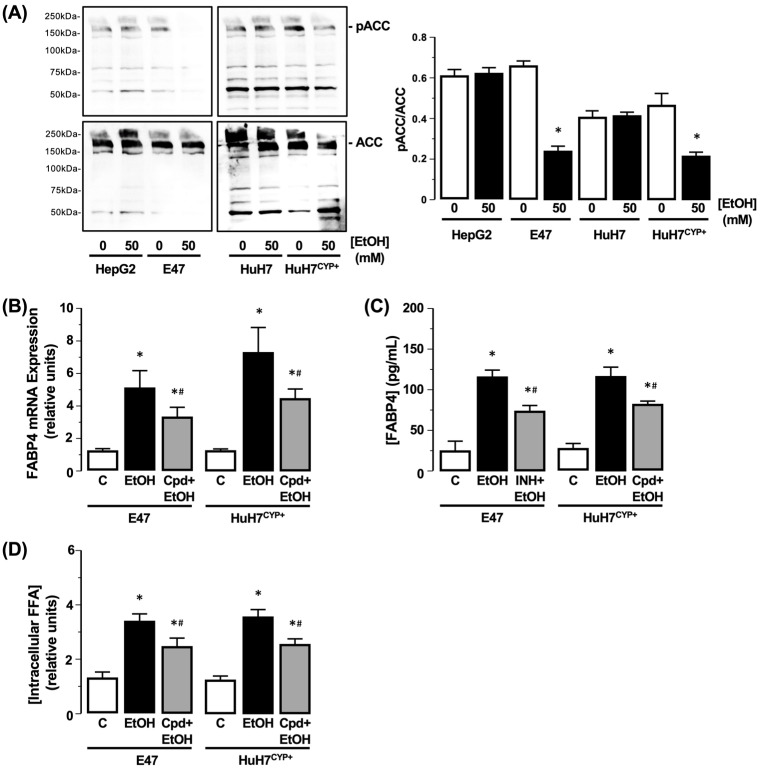
Effect of CYP2E1-EtOH metabolism on ACC activity, *FABP4* expression, and FFA accumulation. (**A**) Representative Western blot analysis of phospho-ACC (pACC; inactive) and total ACC (active) in HepG2 and HuH7 cells, and cells transfected to express CYP2E1 (E47 and HuH7^CYP+^) following EtOH exposure (50 mM). Protein detection is expressed as ratio of pACC:ACC. C = 0 mM EtOH. N = 3, * *p* < 0.05 EtOH vs. C. Effect of EtOH (50 mM) on E47 and HuH7^CYP+^ cells in the absence or presence of Cpd9 (Cpd; 20 nM) on (**B**) *FABP4* mRNA and (**C**) *FABP4* protein in the culture medium, and (**D**) intracellular FFA. N = 3, * *p* < 0.05 EtOH vs. C, ^#^ *p* < 0.05 Cpd9 + EtOH vs. EtOH.

Using the parent HepG2 and HuH7 cell lines, total and phosphorylated ACC (ACC/pACC) were detected, and levels of both forms were unchanged following EtOH exposure (50 mM) compared to the control (0 mM EtOH) (Figure 3A). In contrast, using CYP2E1-expressing cells, levels of pACC detected in both the E47 and HuH7^CYP+^ cells decreased following EtOH exposure (50 mM) compared to the control (0 mM EtOH) (Figure 3A, N = 3, * *p* < 0.05 EtOH vs. Control). To evaluate the potential role of ACC-pACC in regulating *FABP4* and steatosis, cells were cultured with Cpd9 (20nM) prior to EtOH exposure (50 mM). In this setting, *FABP4* mRNA and protein levels remained elevated in the presence of EtOH, albeit to a lesser degree than observed for EtOH alone (Figure 3B,C, N = 3, * *p* < 0.05 EtOH vs. Control, ^#^ *p* < 0.05 EtOH vs. Cpd9 + EtOH). Analysis of intracellular FFA demonstrated Cpd9 failed to prevent EtOH-dependent increases in steatosis (Figure 3D, N = 3, * *p* < 0.05 EtOH vs. Control, ^#^ *p* < 0.05 EtOH vs. Cpd9 + EtOH).

#### 3.3.2. Lipin-1β

**Figure 4:**
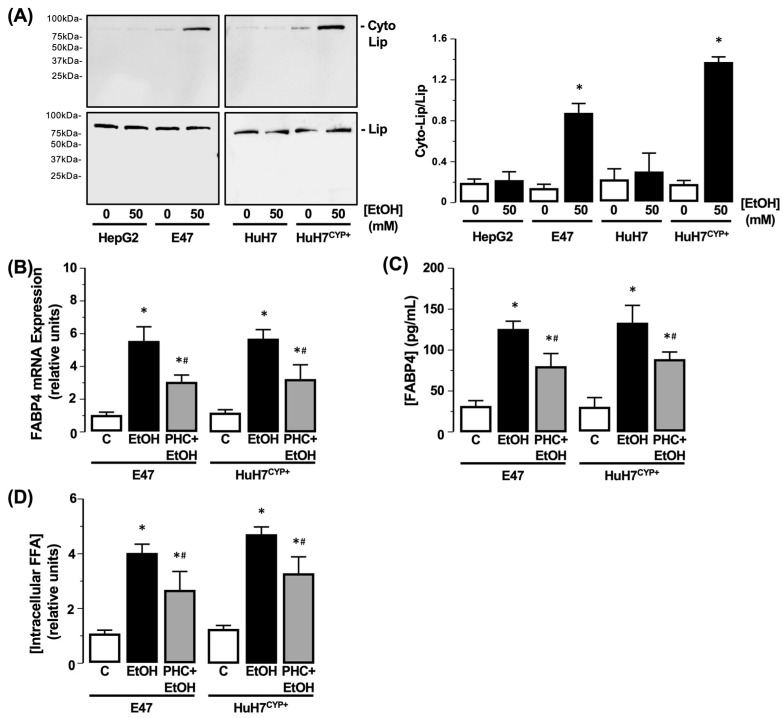
Effect of CYP2E1-EtOH metabolism on Lipin-1β localization and activity, *FABP4* expression, and FFA accumulation. (**A**) Representative Western blot analysis of cytoplasmic Lipin-1β (Cyto-Lip) and total Lipin-1β (Lip) detected in HepG2 and HuH7 cells, and cells transfected to express CYP2E1 (E47 and HuH7^CYP+^), following EtOH exposure (50 mM). Protein detection is expressed as a ratio of Cyto-Lip:LipC. C = 0 mM EtOH. N = 3, * *p* < 0.05 EtOH vs. C. Effect of EtOH (50 mM) on E47 and HuH7^CYP+^ cells in the absence or presence of propranolol hydrochloride (PHC; 10 mM) on (**B**) *FABP4* mRNA, (**C**) *FABP4* protein in culture medium, and (**D**) intracellular FFA. N = 3, * *p* < 0.05 EtOH vs. C, ^#^ *p* < 0.05 PHC + EtOH vs. EtOH.

Using HepG2 and HuH7 cells and E47 and HuH7^CYP+^ cells, total Lipin-1β detection was unchanged after EtOH exposure (50 mM) compared to the control (0 mM EtOH) (Figure 4A). Analysis of the cytoplasmic fraction preparation demonstrated robust detection of GAPDH in the absence of histone 3 detection (Supplemental Figure S3). Using the cytoplasmic fraction preparation, subsequent Western blot analysis demonstrated no change in cytoplasmic Lipin-1β detected in HepG2 or HuH7 cells following EtOH exposure. Conversely, cytoplasmic Lipin-1β markedly increased in E47 and HuH7^CYP+^ cells following EtOH exposure compared to the control (0 mM EtOH) (Figure 4A, N = 3, * *p* < 0.05 EtOH vs. control). To evaluate the potential role of Lipin-1β in regulating *FABP4* expression and steatosis, cells were cultured in the absence or presence of PHC (10 µM) prior to EtOH exposure (50 mM). Using this approach, *FABP4* mRNA and protein levels remained elevated in the presence of EtOH, albeit to a lesser degree than observed for EtOH alone (Figure 4B,C, N = 3, * *p* < 0.05 EtOH vs. control, ^#^ *p* < 0.05 EtOH vs. PHC + EtOH). Analysis of intracellular FFA demonstrated that PHC failed to prevent EtOH-dependent increases in steatosis (Figure 4D, N = 3, * *p* < 0.05 EtOH vs. control, ^#^ *p* < 0.05 EtOH vs. PHC + EtOH).

#### 3.3.3. Sterol Regulatory-Element Binding Protein (SREBP-1c)

**Figure 5:**
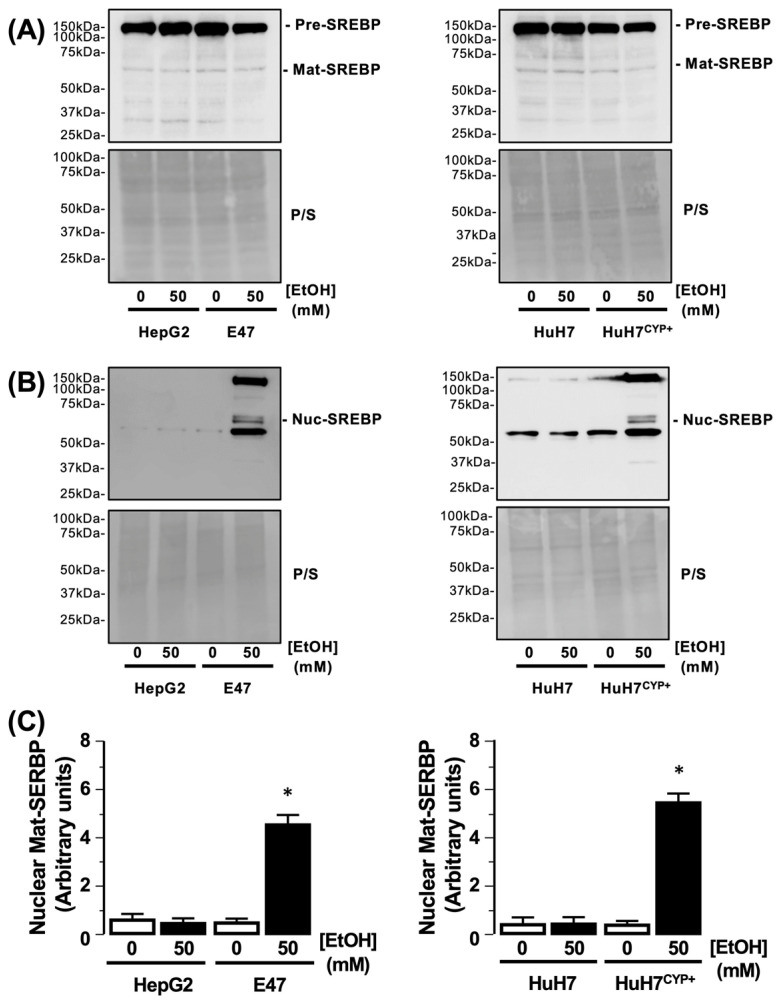
Effect of CYP2E1-EtOH metabolism on SREBP-1c activity and localization. (**A**) Representative Western blot analysis of precursor and mature SREBP1c (Pre-SREBP-1c/mat-SREBP1c) detected in HepG2 and HuH7 cells, and cells transfected to express CYP2E1 (E47 and HuH7^CYP+^) following EtOH exposure (50 mM). Equal protein loading was assessed from membrane Ponceau S staining (P/S). (**B**) Representative Western blot analysis of mat-SREBP-1C detected in nuclear fractions from HepG2/HuH7 and E47/HuH7^CYP+^ cells following EtOH exposure (50 mM). Equal protein loading was assessed from membrane P/S staining. (**C**) Cumulative densitometric analysis of Mat-SREBP-1C detected in nuclear fractions following EtOH exposure (50 mM) and corrected for protein loading (P/S). N = 3, * *p* < 0.05, 50 mM EtOH vs. 0 mM EtOH.

Using HepG2 and HuH7 cells, both the precursor and mature SREBP-1c forms were detected in cell lysates, and detection of both forms was not different following exposure to EtOH (50 mM) (Figure 5A). Similarly, both forms of SREBP-1c were detected in total cell lysates from E47 and HuH7^CYP+^, and detection of both forms was not different following exposure to EtOH (50 mM) (Figure 5A). Analysis of the nuclear fraction preparation demonstrated robust detection of histone 3 in the absence of GAPDH detection (Supplemental Figure S3). Using the nuclear fraction preparation, subsequent Western blot analysis demonstrated that no differences were detected for the HepG2 and HuH7 cells in the presence of EtOH (Figure 5B). Conversely, increased nuclear SREBP-1c was detected in nuclear fractions prepared from both E47 and HuH7^CYP+^ cells following EtOH exposure compared to the control (0 mM EtOH) (Figure 5B, N = 3, * *p* < 0.05 nuclear SREBP-1c control vs. nuclear SREBP-1c + 50 mM EtOH).

**Figure 6:**
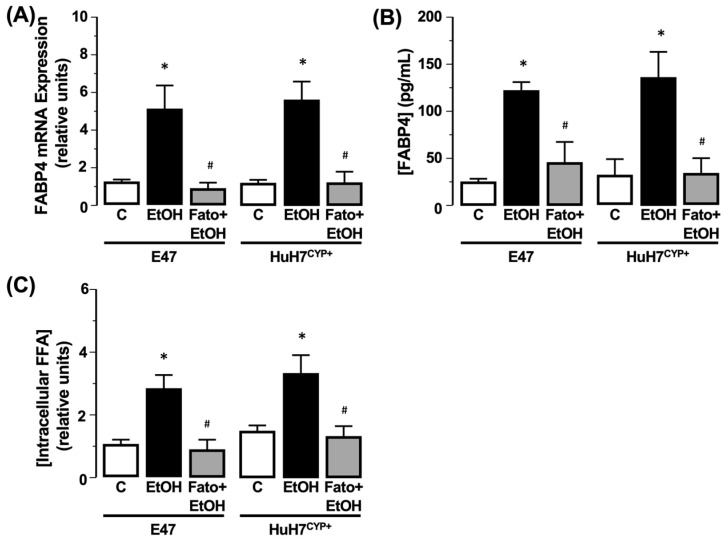
Inhibition of SREBP-1C abrogates the effect of EtOH on *FABP4* expression and FFA accumulation in CYP2E1 expressing cells. Effect of EtOH (50 mM) in cells transfected to express CYP2E1 (E47 and HuH7^CYP+^) in the presence of fatostatin (Fato; 10 µM) on (**A**) *FABP4* mRNA expression, (**B**) *FABP4* protein in the culture medium, and (**C**) intracellular FFA. C = 0 mM EtOH. N = 3, * *p* < 0.05 EtOH vs. C, ^#^ *p* < 0.05 Fato + EtOH vs. EtOH.

To evaluate the potential role of SREBP-1c in regulating *FABP4* and steatosis, cells were cultured in the presence of fatostatin (10 µM) prior to EtOH exposure (50 mM). Under these conditions, the increases in *FABP4* mRNA expression and *FABP4* protein in the culture medium caused by EtOH were abolished by fatostatin (Figure 6A, B, N = 3, * *p* < 0.05 EtOH vs. control, ^#^ *p* < 0.05 fatostatin + EtOH vs. EtOH). Analysis of intracellular FFA demonstrated that fatostatin abrogated the effect of EtOH on cellular FFA accumulation (Figure 6C, N = 3, * *p* < 0.05 EtOH vs. control, *p* < 0.05 EtOH vs. fatostatin + EtOH).

## 4. Discussion

A focal point during the development and progression of ALD is the change in the hepatic REDOX state arising due to sustained EtOH metabolism, leading to inhibition of fatty acid β-oxidation and de novo lipogenesis [22]. While this initially manifests as simple steatosis, sustained, heavy alcohol ingestion leads to persistent chemical and metabolic pressure to promote immune cell infiltration, cell damage, and cell death/replacement [1,3]. Numerous studies have addressed an array of biochemical pathways that are subject to dysregulation in hepatocytes following EtOH metabolism. As these reports highlight, while critical focal points of dysregulation arise, multiple downstream effector pathways exist that directly or indirectly contribute to net hepatocyte lipid content [23,24].

**Figure 7:**
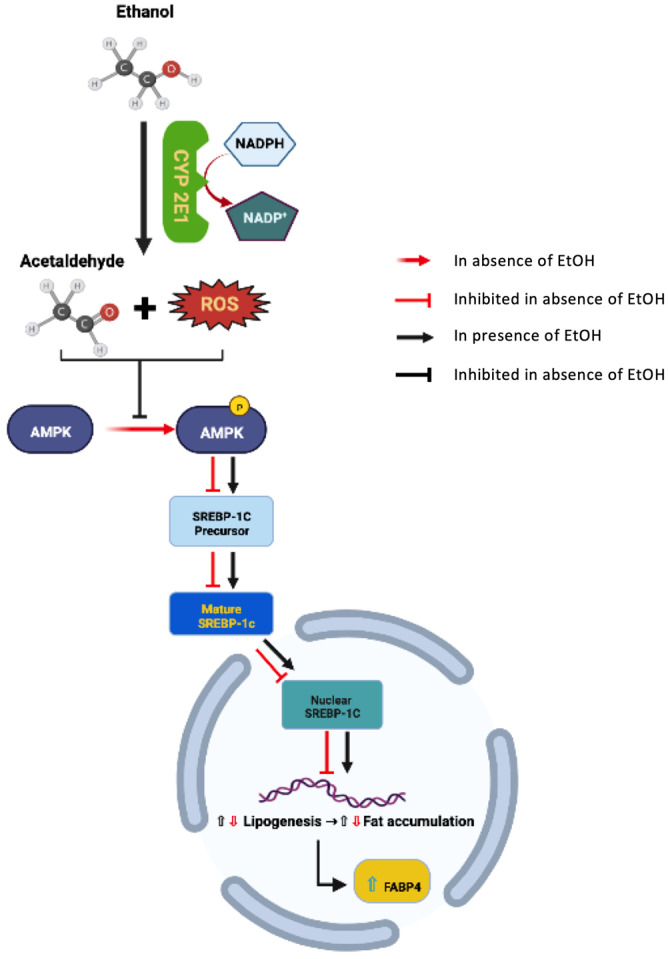
Potential mechanisms regulating hepatic *FABP4* expression following CYP2E1-EtOH metabolism. In normal liver, AMPK is phosphorylated (pAMPK) to prevent maturation and nuclear localization of SREBP-1c. Doing so promotes fatty acid oxidation and reduces lipogenesis in order to maintain intracellular lipid balance. Sustained EtOH exposure leads to CYP2E1 induction, increased acetaldehyde production, and generation of reactive oxygen species (ROS). This increase in intracellular acetaldehyde/ROS blocks AMPK-phosphorylation and promotes the maturation and nuclear localization of SREBP-1C. In doing so, nuclear SREBP-1c alters transcription factor regulation to reduce fatty acid oxidation and enhance lipogenesis. This net intracellular fatty acid accumulation may induce *FABP4* mRNA expression and *FABP4* protein production/secretion.

The focus of this study was the impact of EtOH metabolism on AMPKα activity (and downstream effector pathways), hepatosteatosis, and subsequent changes in hepatic FABP4. Increased intracellular ROS and acetaldehyde following CYP2E1-dependent EtOH metabolism inhibits AMPKα-phosphorylation (activation) and promotes hepatocyte transition from a catabolic to an anabolic state via activation of energy (ATP) dependent pathways, including mechanisms that regulate de novo lipogenesis [25] (Figure 7).

This was evidenced in our studies, whereby a pronounced decrease in pAMPKα was detected in CYP2E1-expressing cells following EtOH exposure. Subsequent analysis of down-stream targets of pAMPKα identified similar changes in Lipin-1β, ACC, and SREBP-1c activity/localization to those reported by other investigators [24,26,27,28,29]. Using pharmacological agents, we report that inhibition of ACC/pACC or Lipin-1β blunted the effect of EtOH on *FABP4* production and intracellular FFA accumulation in CYP2E1-expressing cells. However, pharmacological inhibition of SREBP-1c signaling (fatostatin) abolished the effects of CYP2E1-EtOH metabolism on FFA accumulation and *FABP4* expression. When interpreting these data, it should be highlighted that these studies were performed using a single dose of pharmacological inhibitors which was previously reported by other investigators to be efficacious, and studies using a broader range of inhibitor concentrations may reveal further reductions in FFA accumulation and/or *FABP4* expression. Similarly, the use of a combination of inhibitors may prove useful in identifying the role of pathway interactions versus individual signaling pathways. In addition, given the potential for off-target/non-specific effects of pharmacological agents, our data suggest that future studies using molecular biology approaches to silence specific components AMPK signaling pathways, with or without pharmacological agents, could reveal further details of the mechanisms by which CYP2E1-EtOH metabolism regulates hepatic FFA and/or *FABP4* levels.

Intracellularly, SREBPs are synthesized as inactive precursors that are inserted into the endoplasmic reticulum and nuclear membranes [30]. Depletion of intracellular sterols (e.g., cholesterol) leads to SREBP-1c precursor transportation to the Golgi [31] as well as cleavage to an active/mature SREBP-1c. Following nuclear translocation, binding of mature- SREBP-1c to sterol regulatory element (SRE) occurs on multiple genes which encode proteins that promote triglyceride/cholesterol synthesis and lipid uptake [32]. Conversely, pAMPKα attenuates hepatic steatosis by phosphorylating Ser372 on SREBP-1c in order to inhibit cleavage of precursor SREBP-1c to a transcriptionally active, mature SREBP-1c [33].

In considering these data, we report that, in conjunction with CYP2E1-EtOH metabolism altering AMPKα-SREBP-1c dependent intracellular lipid accumulation, *FABP4* mRNA expression increased in parallel with increased *FABP4* protein detection in the culture medium. To date, most of the work studying FABPs has focused on their role as intracellular FFA chaperones during lipid mobilization and storage. Within the liver, *FABP1* is the predominant FABP detected in hepatocytes [4]. However, previous studies by our group report that in hepatosteatotic mouse models of ALD and NAFLD, *FABP1* mRNA expression is unchanged compared to mice maintained on control diets [6]. These data suggest the induction of *FABP4* mRNA, and de novo *FABP4* protein synthesis (in response to cellular steatosis) may act as a compensatory mechanism to account for a lack in increased FABP1, but does not account for elevated *FABP4* in the cell culture medium.

Physiologically, *FABP4* is most readily detected in adipocytes and macrophages, and significant work has been performed in order to understand its role in these cell types. For example, in addition to binding FFAs, *FABP4* can form functional nuclear localization and nuclear export signals after binding ligands to influence gene transcription [34]. Of particular note, several studies have reported that adipocyte-derived *FABP4* in the setting of metabolic disorders acts as a paracrine-endocrine signaling agent in the development and/or progression of vascular disease [8]. Similarly, several authors have reported that tumors arising within or adjacent to adipose tissue exhibit altered *FABP4* expression. For example, studies using ovarian cancer cells indicate increased *FABP4* expression in tumor cells located adjacent to adipocytes [35]. Equally, *FABP4* secreted by periprostatic adipose tissue is taken up by prostate cancer cells in order to promote tumor invasiveness, while metastatic prostate cancer cells exposed to adipocyte-conditioned media demonstrate increased hemeoxygenase-1, IL-1β expression and increased tumor cell invasiveness, an effect attenuated by blocking *FABP4* or IL-1β signaling [36,37,38].

Within the liver, the potential role of *FABP4* in hepatic pathology remains to be fully elucidated. Previous studies have reported increased *FABP4* mRNA expression and *FABP4* protein in the culture medium using in vitro models of EtOH- and fat-induced cell steatosis, and in animal models of ALD and NAFLD, increased hepatic *FABP4* mRNA and serum *FABP4*. These effects are mirrored in the human liver as well as serum samples from patients with ALD or NAFLD [6]. Additionally, exposure of human hepatoma cells to exogenous *FABP4* stimulates FFA accumulation, cell proliferation, and cell migration [7]. Conversely, a study by Zhong and colleagues reports that patients diagnosed with HCC in the setting of underlying viral hepatitis B (HBV^+^) infection exhibit low *FABP4* expression in HCC tissue, and that the level of *FABP4* expression is associated with tumor size and overall survival. Furthermore, overexpressing *FABP4* in hepatic tumor cell lines inhibited tumor expansion in an ectopic mouse model in vivo [39]. Of interest, when comparing the differences between these studies, it is noteworthy that in Zhong’s report, patients had underlying HBV infection and the cell lines were derived from HBV^+^ patients. In contrast, the cell lines we employed were HBV^-^, and our previous studies report that increased *FABP4* expression was only detected in patients with ALD or NAFLD, and not those with an underlying HBV/HCV infection.

These findings, using samples from disparate patient populations, certainly warrant further investigation using larger sample sizes to allow a greater degree of subgroup analysis. However, the striking differences in the effect of *FABP4* between cell populations suggests that the role of *FABP4* in tumor progression may depend on the underlying risk factor in which cell transformation occurs, as well as the underlying pathology in which the tumor progresses. This may have relevance in the setting of ALD and NAFLD, where hepatosteatosis is present as an early pathology in most patients. For example, in the setting of ALD, the deleterious effects of increased acetaldehyde and ROS resulting from CYP2E1-dependent EtOH metabolism on intracellular protein, lipid, and nucleic acid structure and function are well reported, and underlie the increased rates of hepatocyte turnover and transformation in ALD [1,3,10]. This raises the intriguing possibility that as hepatocytes adapt to sustained fat accumulation through increased *FABP4* production and release, adjacent cells that undergo transformation (due to genetic damage induced by acetaldehyde and ROS) may undergo accelerated rates of growth and migration due to increased intrahepatic *FABP4* released from steatotic hepatocytes.

In addition to the potential role of *FABP4* as a pro-tumorigenic autocrine/paracrine signaling molecule, further work is also required to understand the impact of increased intracellular *FABP4* on cellular homeostasis. For example, increased intracellular *FABP4* expression has the potential to alter both the amount of lipid accumulation within the cell and the specific type of lipids that are stored. Previous studies indicate an integral role for FABP1 in regulating cholesterol uptake and cellular distribution in hepatocytes [40,41,42]. Similarly, *FABP4* is reported to enhance cholesterol uptake/accumulation in macrophages [43]. This may have particular significance in the setting of ALD given the integral role of SREBP-1 as an intracellular sensor of cholesterol levels [23,24,26] and the impact of EtOH-metabolism on SREBP-1 expression/localization [30,31,32]. Equally, a change in the balance of pathways that regulate FFA sequestration, transport, and accumulation is also likely to impact other pathways that regulate the metabolic homeostasis of hepatic cells, including glucose homeostasis. Indeed, this may be of particular interest for future studies, given the complex relationship between chronic alcohol ingestion, steatosis, insulin resistance, and progression of ALD [44,45].

When considering the data presented in this study, several potential limitations should be highlighted. Primarily, the focus of this study was to expand our understanding of potential mechanisms by which EtOH metabolism may alter hepatic *FABP4* expression and increased intracellular triglyceride accumulation. In order to do so, we employed commercially available human hepatoma cells and derivatives that have been stably transfected to express CYP2E1 or ADH. The transformed nature of these cells makes them fundamentally different to hepatocytes at a genetic, biochemical, and physiological level. Additionally, the in vitro nature of the studies performed is clearly very different to that of hepatocytes in vivo, and there are complexities associated with the intra- and extra-hepatic environment that exists in the development and progression of ALD. Secondly, our studies made extensive use of pharmacological inhibitors. While every effort was made to use compounds previously used by other investigators [18,19,20,21], the use of such an approach runs the risk of off non-specific intracellular effects, and further work using molecular biology approaches (e.g., shRNA/siRNA or CRISPR/Cas9) is required in order to confirm these data. Finally, great care should be taken not to over-interpret these types of in vitro mechanistic data toward the broader potential implications of how *FABP4* may function in the highly complex in vivo pathology of ALD, in which intrahepatic and extrahepatic responses are critical in defining disease progression.

## 5. Conclusions

Our data demonstrate that CYP2E1-EtOH metabolism inhibits AMPKα phosphorylation (activation) and stimulates FFA accumulation, *FABP4* mRNA expression, and *FABP4* protein secretion. Considering previous reports demonstrating elevated *FABP4* expression in rodent models of ALD and human ALD, as well as the stimulation of cell growth and migration of hepatoma cells in vitro following exposure to exogenous FABP, the role of *FABP4* released from steatotic hepatocytes in promoting tumor development in ALD warrants further investigation.

## Data Availability

Not applicable.

## Supplementary Materials

The following supporting information can be downloaded at: https://www.mdpi.com/article/10.3390/biology11111613/s1. 1. Supplemental Figures S1–S3; 2. Complete images of uncropped Western blots and densitometric data used to create data graphs.

## References

[1] McKillop I.H., Schrum L.W., Thompson K.J. (2016). Role of alcohol in the development and progression of hepatocellular carcinoma. Hepatic Oncol..

[2] Subramaniyan V., Chakravarthi S., Jegasothy R., Seng W.Y., Fuloria N.K., Fuloria S., Hazarika I., Das A. (2021). Alcohol-associated liver disease: A review on its pathophysiology, diagnosis and drug therapy. Toxicol. Rep..

[3] Neuman M.G., French S.W., French B.A., Seitz H.K., Cohen L.B., Mueller S., Osna N.A., Kharbanda K.K., Seth D., Bautista A. (2014). Alcoholic and non-alcoholic steatohepatitis. Exp. Mol. Pathol..

[4] Eguchi A., Iwasa M. (2021). The Role of Elevated Liver-Type Fatty Acid-Binding Proteins in Liver Diseases. Pharm. Res..

[5] Thumser A.E., Moore J.B., Plant N. (2014). Fatty acid binding proteins: Tissue-specific functions in health and disease. Curr. Opin. Clin. Nutr. Metab. Care.

[6] Thompson K.J., Austin R.G., Nazari S.S., Gersin K.S., Iannitti D.A., McKillop I.H. (2017). Altered fatty acid-binding protein 4 (FABP4) expression and function in human and animal models of hepatocellular carcinoma. Liver Int..

[7] Attal N., Sullivan M.T., Girardi C.A., Thompson K.J., McKillop I.H. (2020). Fatty acid binding protein-4 promotes alcohol-dependent hepatosteatosis and hepatocellular carcinoma progression. Transl. Oncol..

[8] Rodríguez-Calvo R., Girona J., Alegret J.M., Bosquet A., Ibarretxe D., Masana L. (2017). Role of the fatty acid-binding protein 4 in heart failure and cardiovascular disease. J. Endocrinol..

[9] McKillop I.H., Girardi C.A., Thompson K.J. (2019). Role of fatty acid binding proteins (FABPs) in cancer development and progression. Cell. Signal..

[10] Cederbaum A.I. (2012). Alcohol Metabolism. Clin. Liver Dis..

[11] Seitz H.K. (2020). The role of cytochrome P4502E1 in the pathogenesis of alcoholic liver disease and carcinogenesis. Chem. Biol. Interact..

[12] You M., Jogasuria A., Taylor C., Wu J. (2015). Sirtuin 1 signaling and alcoholic fatty liver disease. Hepatobiliary Surg. Nutr..

[13] Ding R.-B., Bao J., Deng C.-X. (2017). Emerging roles of SIRT1 in fatty liver diseases. Int. J. Biol. Sci..

[14] Bai J., Cederbaum A.I. (2001). Adenovirus-mediated Overexpression of Catalase in the Cytosolic or Mitochondrial Compartment Protects against Cytochrome P450 2E1-dependent Toxicity in HepG2 Cells. J. Biol. Chem..

[15] Donohue T.M., Osna N.A., Clemens D.L. (2006). Recombinant Hep G2 cells that express alcohol dehydrogenase and cytochrome P450 2E1 as a model of ethanol-elicited cytotoxicity. Int. J. Biochem. Cell Biol..

[16] Osna N.A., White R.L., Krutik V.M., Wang T., Weinman S.A., Donohue T.M. (2008). Proteasome Activation by Hepatitis C Core Protein Is Reversed by Ethanol-Induced Oxidative Stress. Gastroenterology.

[17] Attal N., Marrero E., Thompson K.J., McKillop I.H. (2022). Cytochrome P450 2E1-dependent hepatic ethanol metabolism induces fatty acid-binding protein 4 and steatosis. Alcohol. Clin. Exp. Res..

[18] Guo J., Fang W., Chen X., Lin Y., Hu G., Wei J., Zhang X., Yang C., Li J. (2018). Upstream stimulating factor 1 suppresses autophagy and hepatic lipid droplet catabolism by activating mTOR. FEBS Lett..

[19] Li X., Chen Y.-T., Hu P., Huang W.-C. (2014). Fatostatin Displays High Antitumor Activity in Prostate Cancer by Blocking SREBP-Regulated Metabolic Pathways and Androgen Receptor Signaling. Mol. Cancer Ther..

[20] Griffith D.A., Kung D.W., Esler W.P., Amor P.A., Bagley S.W., Beysen C., Carvajal-Gonzalez S., Doran S.D., Limberakis C., Mathiowetz A.M. (2014). Decreasing the Rate of Metabolic Ketone Reduction in the Discovery of a Clinical Acetyl-CoA Carboxylase Inhibitor for the Treatment of Diabetes. J. Med. Chem..

[21] Farah B.L., Sinha R.A., Wu Y., Singh B.K., Zhou J., Bay B.-H., Yen P.M. (2014). β-Adrenergic Agonist and Antagonist Regulation of Autophagy in HepG2 Cells, Primary Mouse Hepatocytes, and Mouse Liver. PLoS ONE.

[22] Rasineni K., Casey C.A. (2012). Molecular mechanism of alcoholic fatty liver. Indian, J. Pharmacol..

[23] Crabb D.W., Liangpunsakul S. (2006). Alcohol and lipid metabolism. J. Gastroenterol. Hepatol..

[24] You M., Arteel G.E. (2019). Effect of ethanol on lipid metabolism. J. Hepatol..

[25] Sid B., Verrax J., Calderon P.B. (2013). Role of AMPK activation in oxidative cell damage: Implications for alcohol-induced liver disease. Biochem. Pharmacol..

[26] You M., Crabb D.W. (2004). Recent Advances in Alcoholic Liver Disease II. Minireview: Molecular mechanisms of alcoholic fatty liver. Am. J. Physiol. Gastrointest Liver Physiol..

[27] Hu M., Wang F., Li X., Rogers C.Q., Liang X., Finck B.N., Mitra M.S., Zhang R., Mitchell D.A., You M. (2011). Regulation of hepatic lipin-1 by ethanol: Role of AMP-activated protein kinase/sterol regulatory element-binding protein 1 signaling in mice. Hepatology.

[28] Zeng T., Zhang C.-L., Song F.-Y., Zhao X.-L., Xie K.-Q. (2014). CMZ Reversed Chronic Ethanol-Induced Disturbance of PPAR-α Possibly by Suppressing Oxidative Stress and PGC-1α Acetylation, and Activating the MAPK and GSK3β Pathway. PLoS ONE.

[29] Chen Y.-Y., Zhang C.-L., Zhao X.-L., Xie K.-Q., Zeng T. (2014). Inhibition of cytochrome P4502E1 by chlormethiazole attenuated acute ethanol-induced fatty liver. Chem. Interactions.

[30] Wang X., Sato R., Brown M.S., Hua X., Goldstein J.L. (1994). SREBP-1, a membrane-bound transcription factor released by sterol-regulated proteolysis. Cell.

[31] Edwards P.A., Tabor D., Kast H.R., Venkateswaran A. (2000). Regulation of gene expression by SREBP and SCAP. Biochim. et Biophys. Acta (BBA)—Mol. Cell Biol. Lipids.

[32] Brown M.S., Goldstein J.L. (1999). A proteolytic pathway that controls the cholesterol content of membranes, cells, and blood. Proc. Natl. Acad. Sci. USA.

[33] Li Y., Xu S., Mihaylova M.M., Zheng B., Hou X., Jiang B., Park O., Luo Z., Lefai E., Shyy J.Y.-J. (2011). AMPK Phosphorylates and Inhibits SREBP Activity to Attenuate Hepatic Steatosis and Atherosclerosis in Diet-Induced Insulin-Resistant Mice. Cell Metab..

[34] Gillilan R.E., Ayers S., Noy N. (2007). Structural Basis for Activation of Fatty Acid-binding Protein 4. J. Mol. Biol..

[35] Nieman K.M., Kenny H.A., Penicka C.V., Ladanyi A., Buell-Gutbrod R., Zillhardt M.R., Romero I.L., Carey M.S., Mills G.B., Hotamisligil G.S. (2011). Adipocytes promote ovarian cancer metastasis and provide energy for rapid tumor growth. Nat. Med..

[36] Herroon M.K., Rajagurubandara E., Hardaway A.L., Powell K., Turchick A., Feldmann D., Podgorski I. (2013). Bone marrow adipocytes promote tumor growth in bone via FABP4-dependent mechanisms. Oncotarget.

[37] Uehara H., Takahashi T., Oha M., Ogawa H., Izumi K. (2014). Exogenous fatty acid binding protein 4 promotes human prostate cancer cell progression. Int. J. Cancer.

[38] Uehara H., Kobayashi T., Matsumoto M., Watanabe S., Yoneda A., Yoshimi B. (2018). Adipose tissue: Critical contributor to the development of prostate cancer. J. Med. Investig..

[39] Zhong C.-Q., Zhang X.-P., Ma N., Zhang E.-B., Li J.-J., Jiang Y.-B., Gao Y.-Z., Yuan Y.-M., Lan S.-Q., Xie D. (2018). FABP4 suppresses proliferation and invasion of hepatocellular carcinoma cells and predicts a poor prognosis for hepatocellular carcinoma. Cancer Med..

[40] Martin G.G., Atshaves B.P., Huang H., McIntosh A.L., Williams B.J., Pai P.-J., Russell D.H., Kier A.B., Schroeder F. (2009). Hepatic phenotype of liver fatty acid binding protein gene-ablated mice. Am. J. Physiol. Gastrointest Liver Physiol..

[41] Favretto F., Assfalg M., Gallo M., Cicero D.O., D’Onofrio M., Molinari H. (2013). Ligand Binding Promiscuity of Human Liver Fatty Acid Binding Protein: Structural and Dynamic Insights from an Interaction Study with Glycocholate and Oleate. ChemBioChem.

[42] Nemecz G., Schroeder F. (1991). Selective binding of cholesterol by recombinant fatty acid binding proteins. J. Biol. Chem..

[43] Wang X.Q., Yang K., He Y.S., Lu L., Shen W.F. (2011). Receptor Mediated Elevation in *FABP4* Levels by Advanced Glycation End Products Induces Cholesterol and Triacylglycerol Accumulation in THP-1 Macrophages. Lipids.

[44] Carr R.M., Dhir R., Yin X., Agarwal B., Ahima R.S. (2013). Temporal Effects of Ethanol Consumption on Energy Homeostasis, Hepatic Steatosis, and Insulin Sensitivity in Mice. Alcohol. Clin. Exp. Res..

[45] Raynard B., Balian A., Fallik D., Capron F., Bedossa P., Chaput J.-C., Naveau S. (2002). Risk factors of fibrosis in alcohol-induced liver disease. Hepatology.

