# Supplementary material for: Role of AMPK-SREBP Signaling in Regulating Fatty Acid Binding-4 (FABP4) Expression following Ethanol Metabolism
Source: Biology (Basel). 2022 Nov 4;11(11):1613. doi: 10.3390/biology11111613 (PMC9687530; doi:10.3390/biology11111613)
Supplement: Supplementary file 1 [file biology-11-01613-s001.zip › Complete images of uncropped Western blots and densitometric data used to create data graphs.pdf]

A)

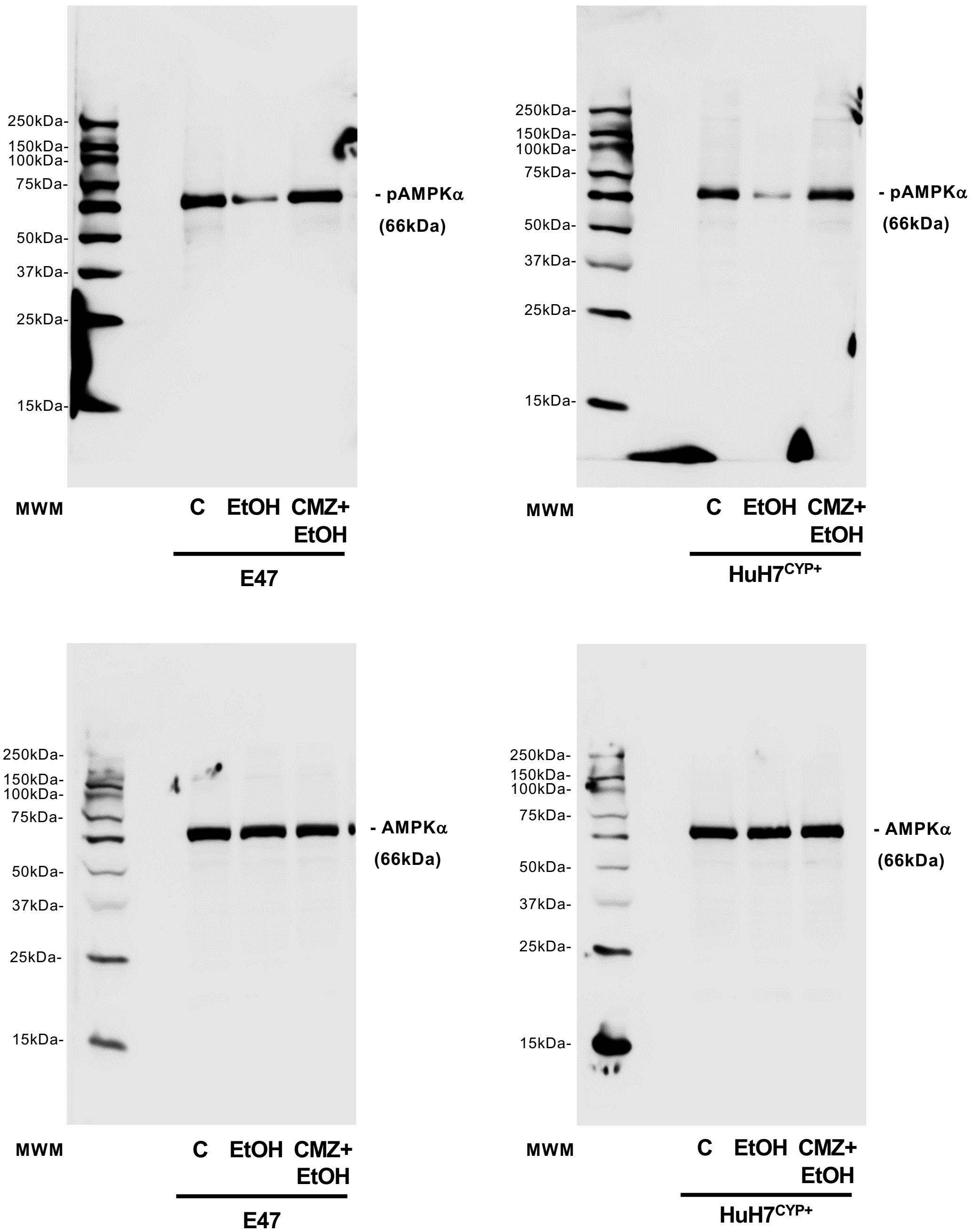

Abbreviations. MWM, Molecular weight marker; C, Control [0mM EtOH], EtOH, 50mM EtOH; CMZ+EtOH, Chlormethiazole (100µM) + EtOH (50mM); E47, HepG2 cells expressing CYP 2E1; $HuH7^{CYP+}$, HuH7 cells expressing CYP 2E1; pAMPKα, phosphorylated AMP-activated protein kinase-α; AMPKα, AMP-activated protein kinase-α

A)

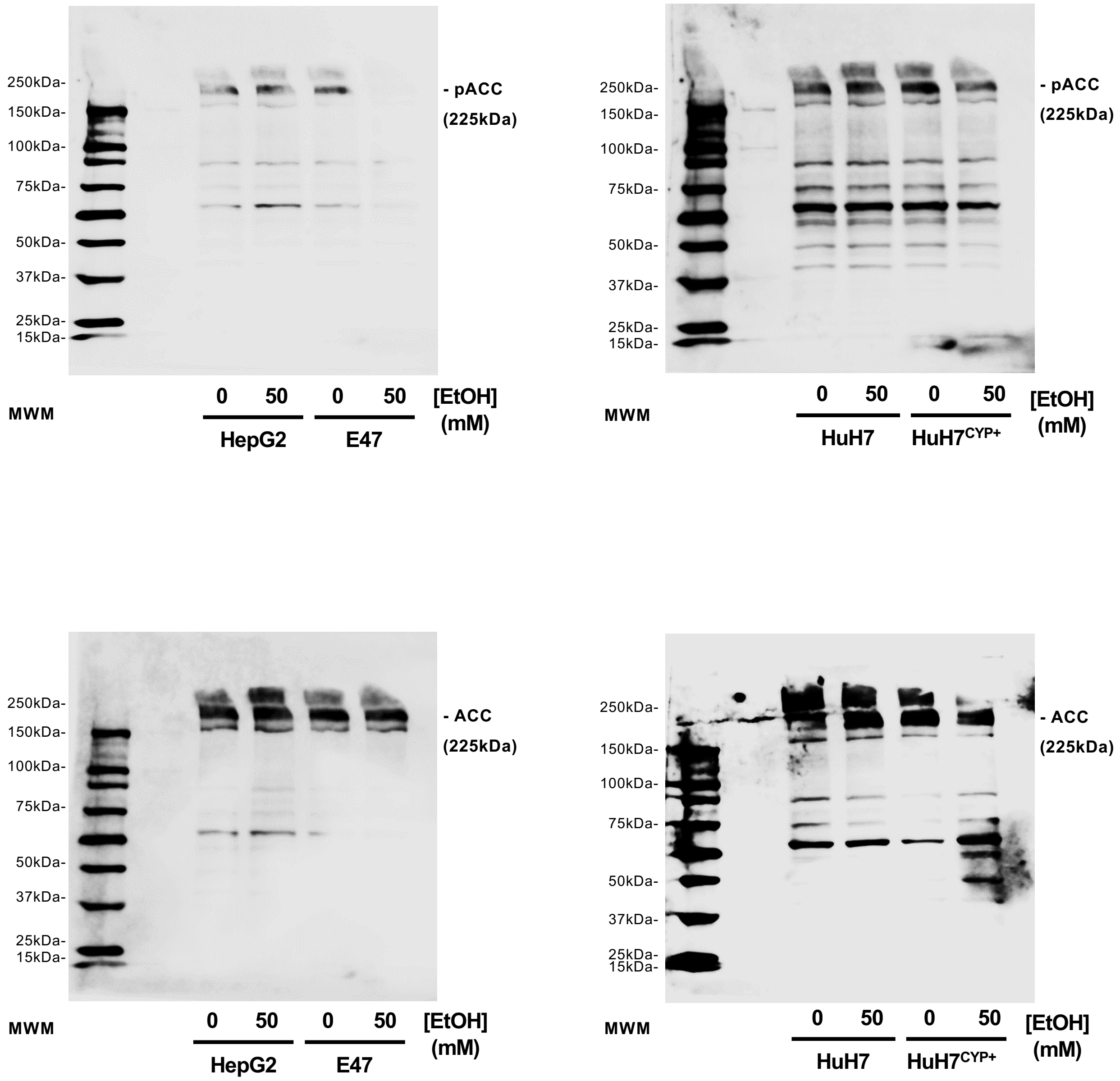

Abbreviations. MWM, Molecular weight marker; E47, HepG2 cells expressing CYP 2E1; HuH7^{CYP+}, HuH7 cells expressing CYP 2E1; pACC, phosphorylated acetyl-CoA carboxylase; ACC, acetyl-CoA carboxylase.

A)

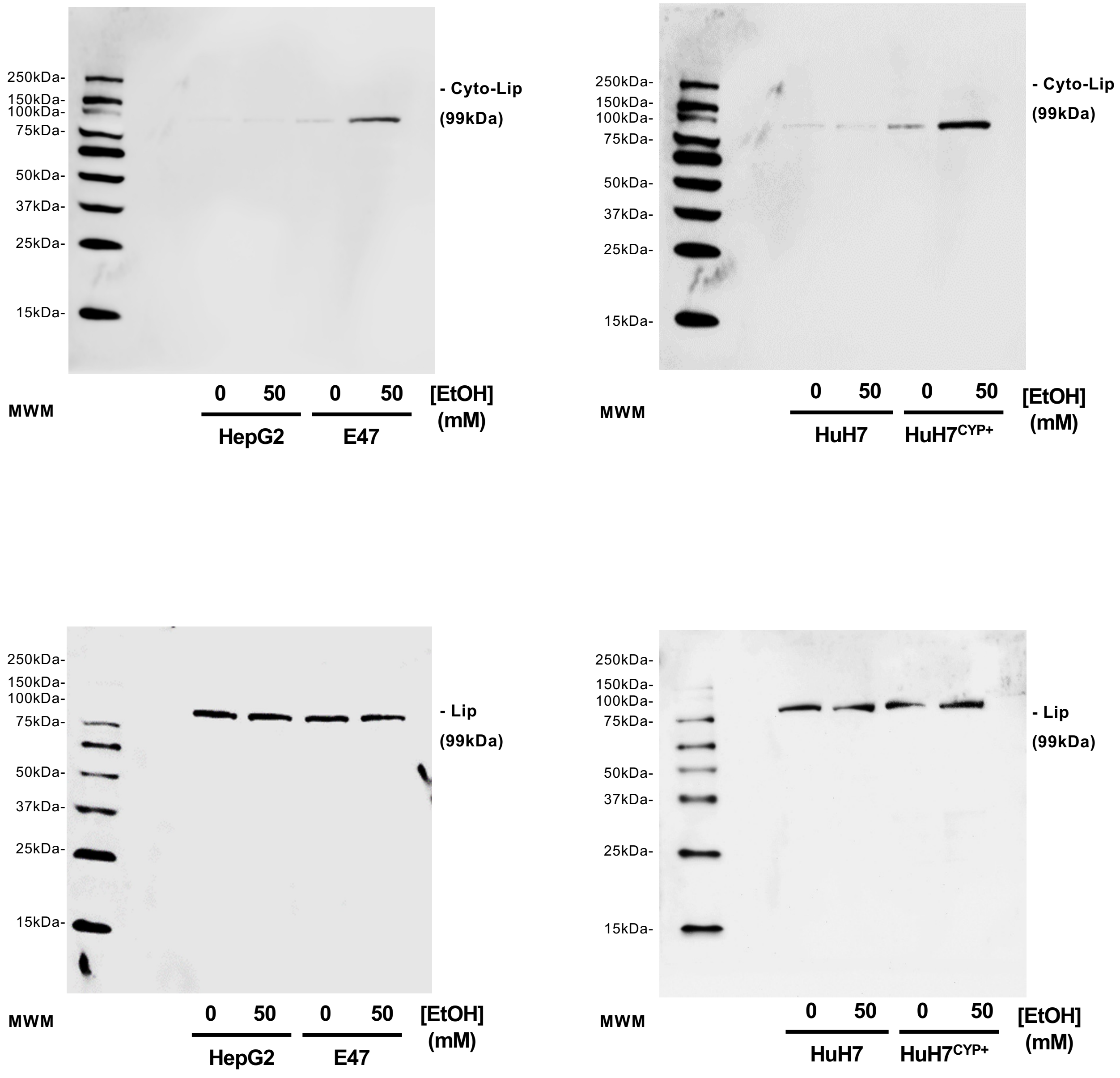

Abbreviations. MWM, Molecular weight marker; E47, HepG2 cells expressing CYP 2E1; HuH7^{CYP+}, HuH7 cells expressing CYP 2E1; Cyto-Lip, Cytoplasmic lipin-1; Lip, Total lipin-1.

A)

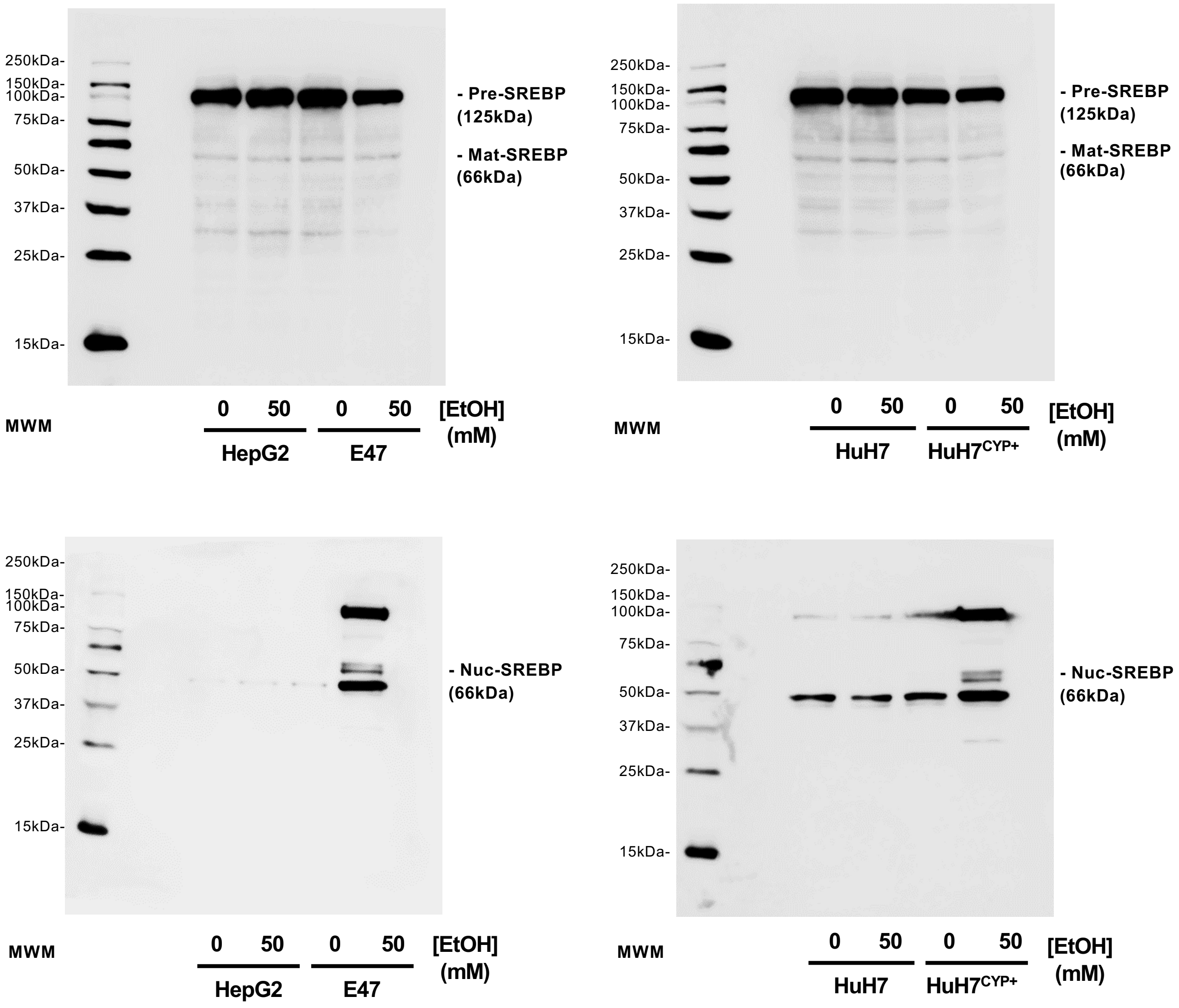

***Abbreviations*.** MWM, Molecular weight marker; E47, HepG2 cells expressing CYP 2E1; HuH7^{CYP+}, HuH7 cells expressing CYP 2E1; Pre-SREBP, Precursor sterol regulatory element binding protein 1c; Mat-SREBP, Mature SREBP; Nub-SREBP, Nuclear SREBP.

A)

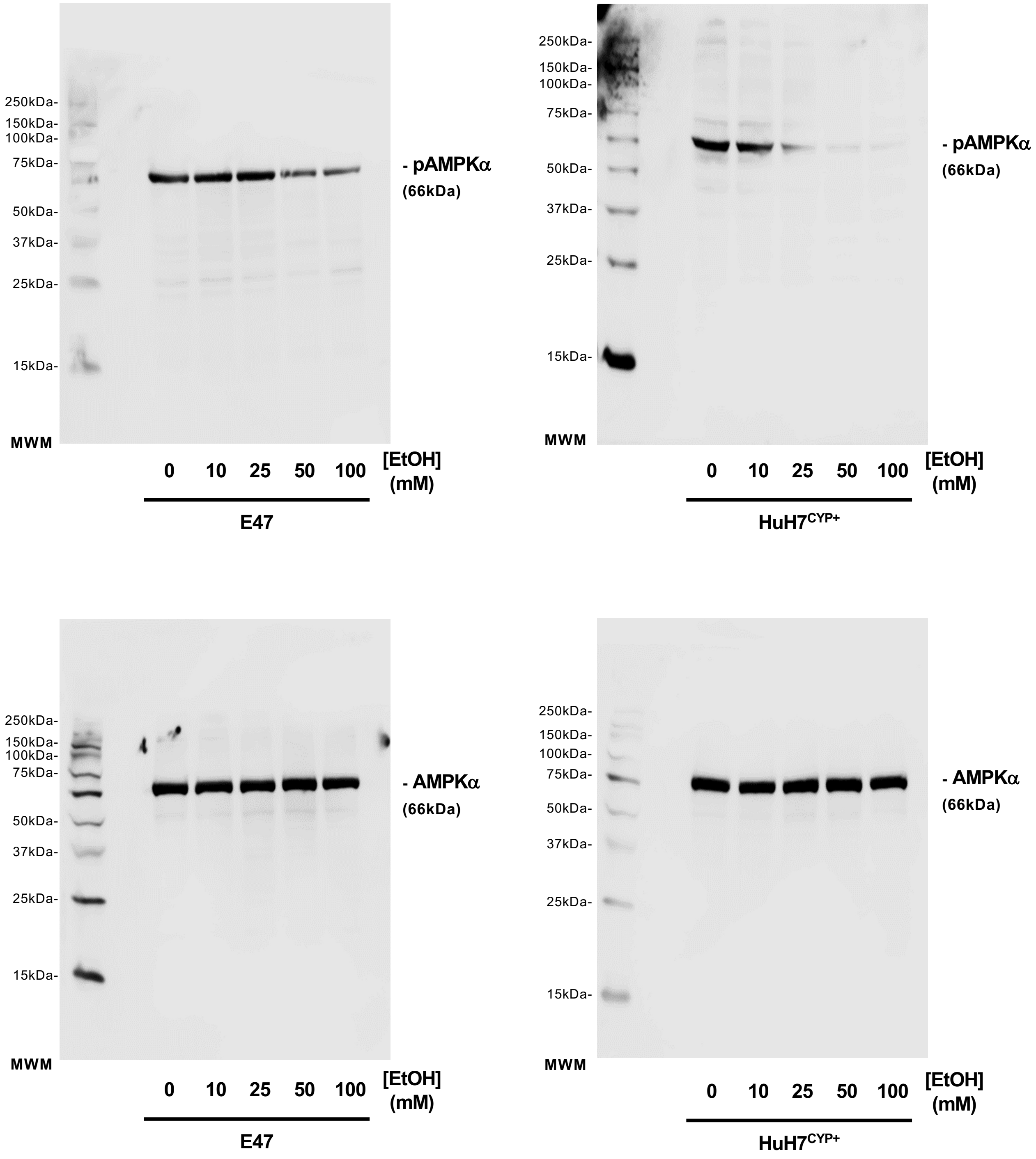

Abbreviations. MWM, Molecular weight marker; E47, HepG2 cells expressing CYP 2E1; HuH7^{CYP+}, HuH7 cells expressing CYP 2E1; pAMPKα, phosphorylated AMP-activated protein kinase-α; AMPKα, AMP-activated protein kinase-α .

A)

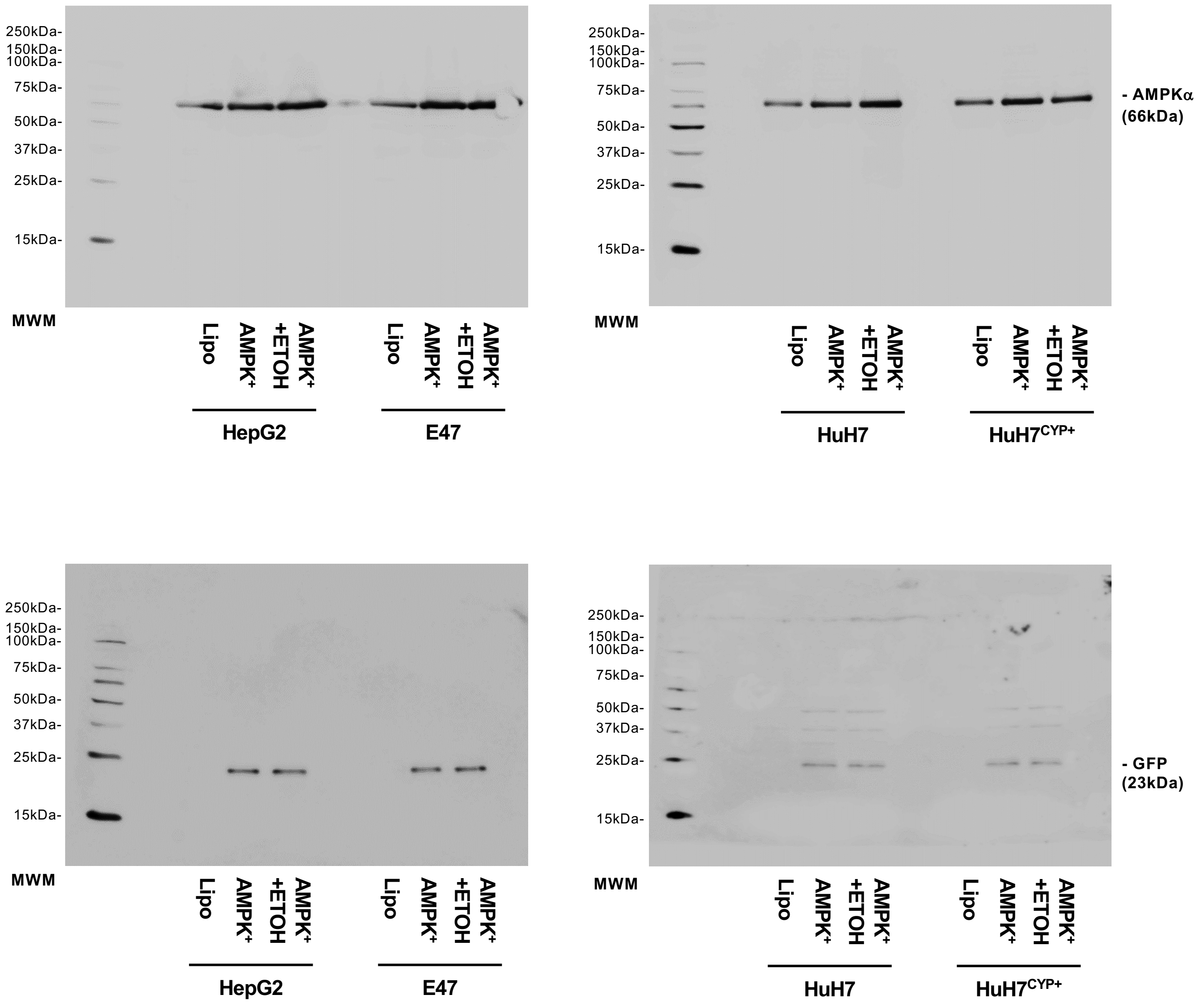

***Abbreviations*.** MWM, Molecular weight marker; E47, HepG2 cells expressing CYP 2E1; HuH7^{CYP+}, HuH7 cells expressing CYP 2E1; Lipo, Lipofectamine only; AMPK^{+}, Cells transfected to express AMP-activated protein kinase-α and green fluorescent protein (GFP); AMPK^{+} + EtOH, Cells transfected to express AMPKα and GFP in the presence of ethanol (EtOH; 50mM).

Uncropped/Full Gel Images

Supplemental Figure S3

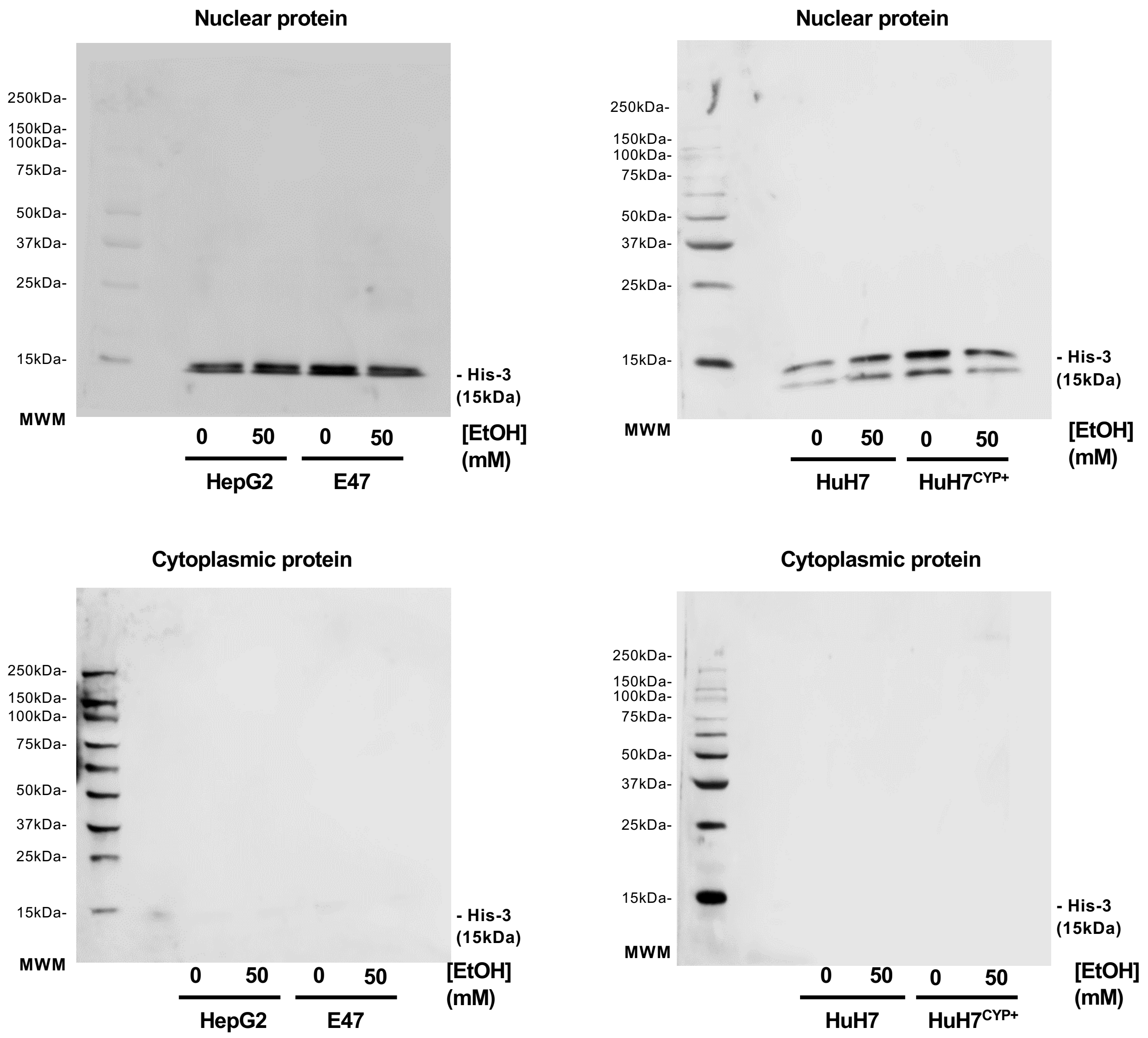

Abbreviations. MWM, Molecular weight marker; E47, HepG2 cells expressing CYP 2E1; HuH7^{CYP+}, HuH7 cells expressing CYP 2E1; His-3, Histone 3 (nuclear protein marker).

Uncropped/Full Gel Images

Supplemental Figure S3

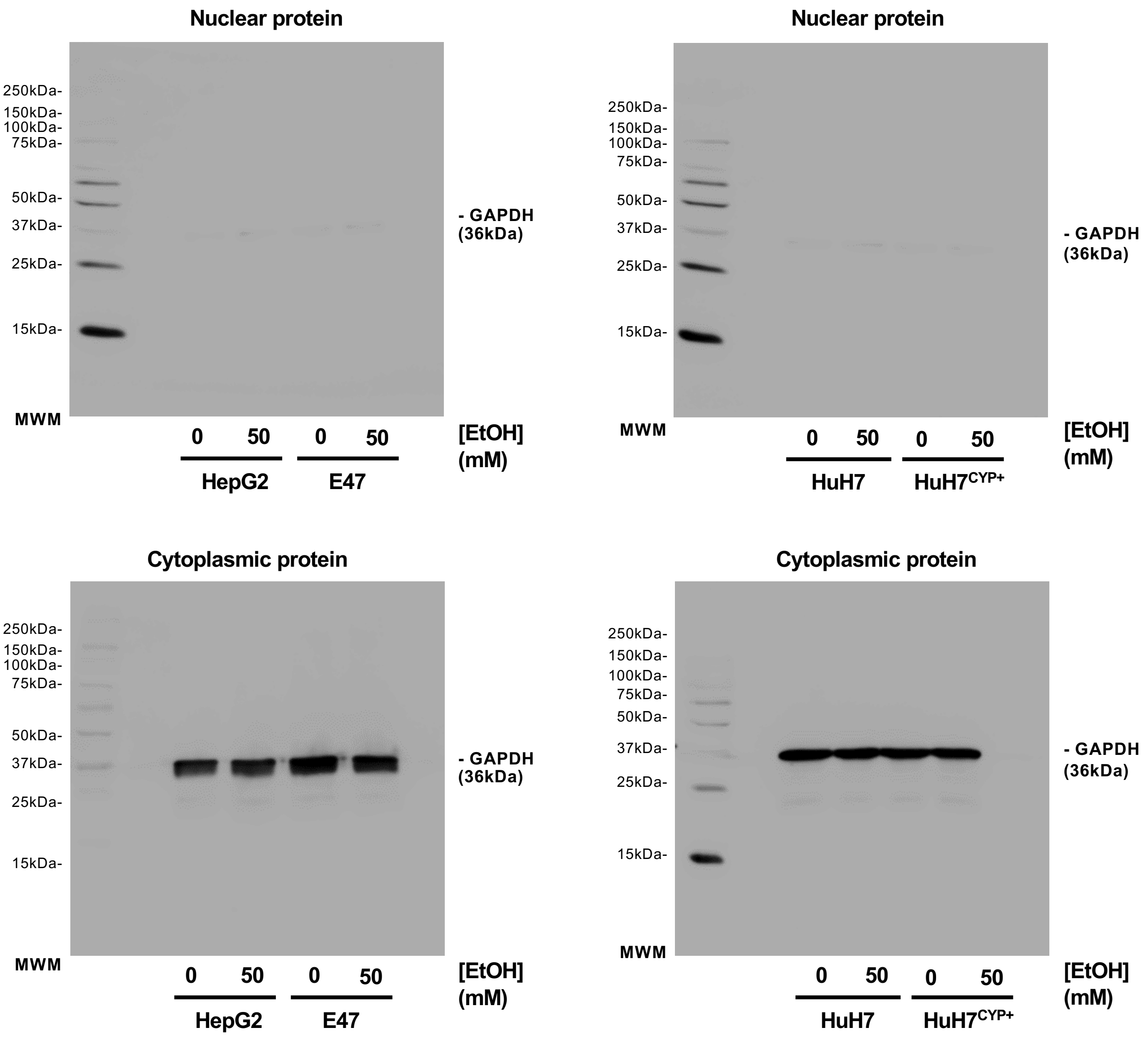

***Abbreviations*.** MWM, Molecular weight marker; E47, HepG2 cells expressing CYP 2E1; HuH7^{CYP+}, HuH7 cells expressing CYP 2E1; GAPDH, Glyceraldehyde-3-phosphate dehydrogenase (cytoplasmic protein marker).

Figure 2A – pAMPK/AMPK ratio

	**0mM EtOH**	**50mM EtOH**	**50mM EtOH + CMZ**
**E47 Cells**	1.101	0.039	1.154
	0.864	0.255	0.730
	0.920	0.082	0.801
**HuH7^{CYP+} Cells**	0.910	0.187	0.760
	0.655	0.127	1.062
	0.512	0.057	0.789

Figure 3A – pACC/ACC ratio

	**0mM EtOH**	**50mM EtOH**
**HepG2 Cells**	0.632	0.655
	0.630	0.526
	0.537	0.650
**E47 Cells**	0.652	0.333
	0.765	0.117
	0.545	0.224
**HuH7 Cells**	0.496	0.470
	0.359	0.344
	0.459	0426
**HuH7CYP+ Cells**	0.603	0.270
	0.421	0.220
	0.399	0.145

Figure 4A – pACC/ACC ratio

	0mM EtOH	50mM EtOH
**HepG2 Cells**	0.047	0.098
	0.293	0.401
	0.153	0.126
**E47 Cells**	0.112	1.102
	0.071	0.768
	0.211	0.736
**HuH7 Cells**	0.420	0.664
	0.119	0.029
	0.051	0.108
**HuH7CYP+ Cells**	0.081	1.501
	0.253	1.282
	0.089	1.271

Figure 5A – Nuclear mature SREBP densitometry (arbitrary units)

	**0mM EtOH**	**50mM EtOH**
**HepG2 Cells**	516.0	234.0
	145.8	156.7
	198.6	334.5
**E47 Cells**	525.1	5145.6
	624.2	4356.9
	210.5	5534.6
**HuH7 Cells**	526.2	545.7
	734.7	834.7
	1056.8	1078.9
**HuH7CYP+ Cells**	456.8	6135.8
	955.0	5515.8
	853.9	4918.9

Supplemental Figure S1A – pAMPK/AMPK ratio

	**[EtOH] (mM)**				
	**0**	**10**	**25**	**50**	**100**
**E47 Cells**	0.863	0.941	0.768	0.424	0.206
	0.779	0.938	1.005	0.289	0.392
	0.828	0.745	0.352	0.097	0.116
**HuH7CYP+ Cells**	0.664	0.621	0.241	0.028	0.016
	0.543	0.447	0.145	0.142	0.081
	0.503	0.478	0.136	0.058	0.012
