# Supplementary figures and images for: Role of AMPK-SREBP Signaling in Regulating Fatty Acid Binding-4 (FABP4) Expression following Ethanol Metabolism
Source: Biology (Basel). 2022 Nov 4;11(11):1613. doi: 10.3390/biology11111613 (PMC9687530; doi:10.3390/biology11111613)

A)

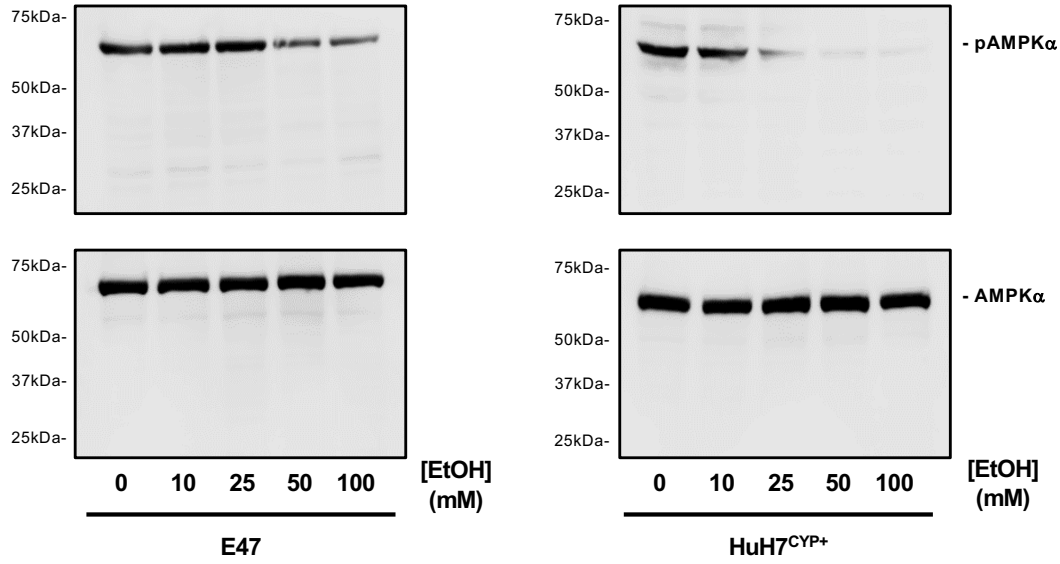

B)

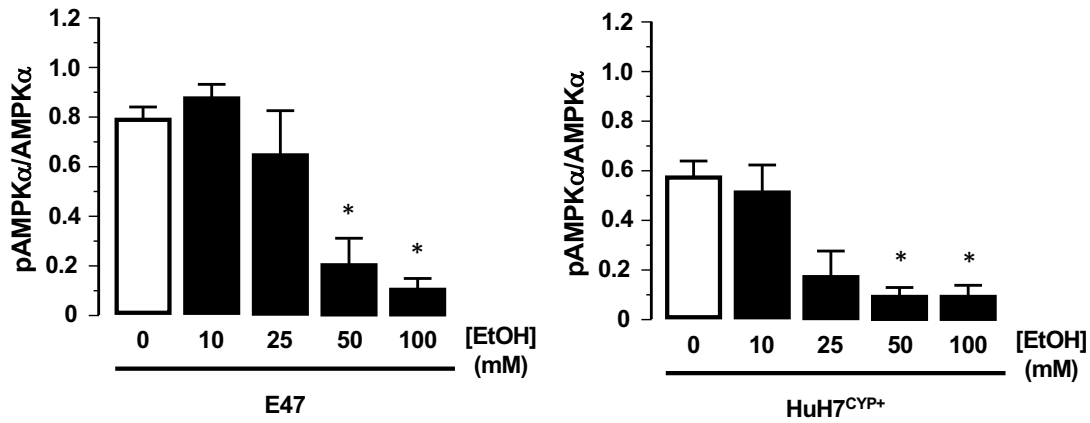

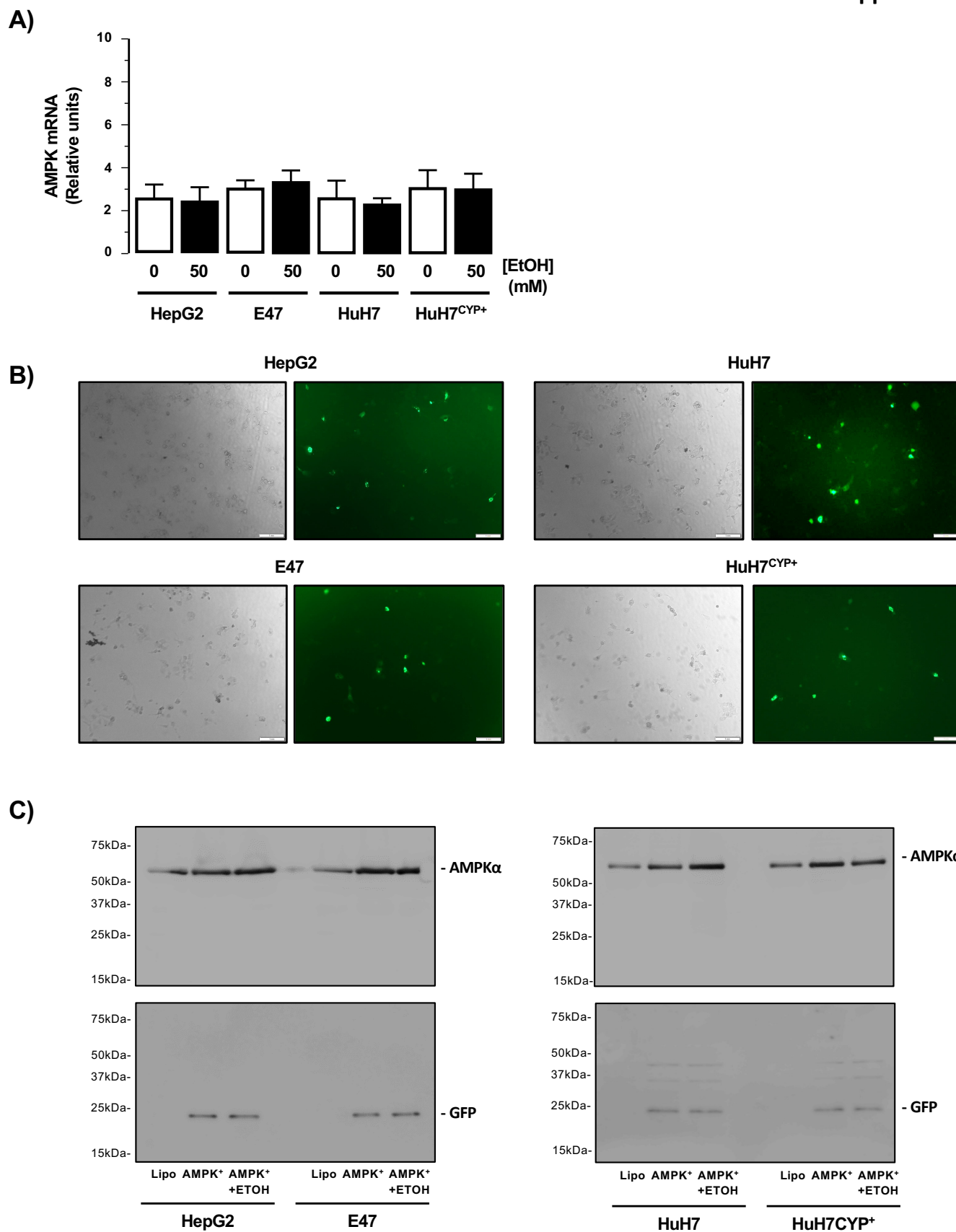

**Nuclear fraction**

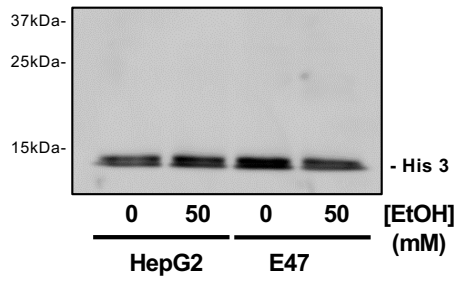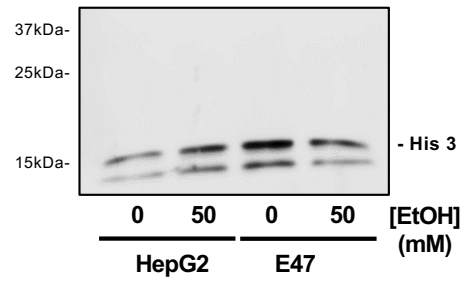

**Cytoplasmic fraction**

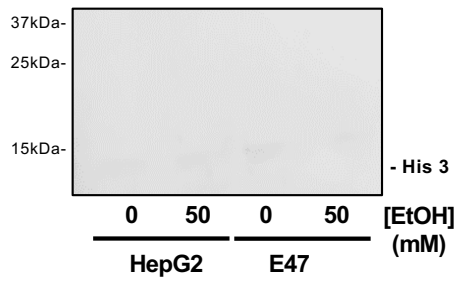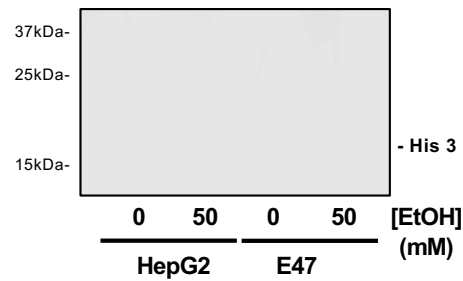

**Nuclear fraction**

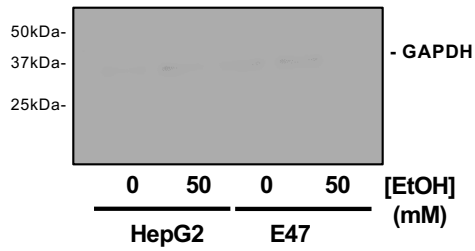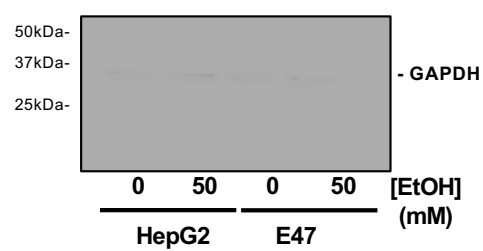

**Cytoplasmic fraction**

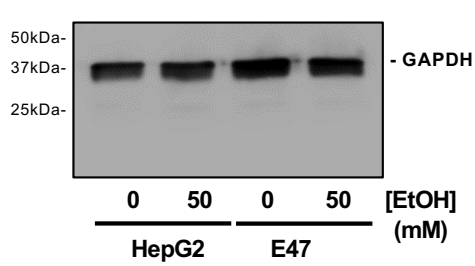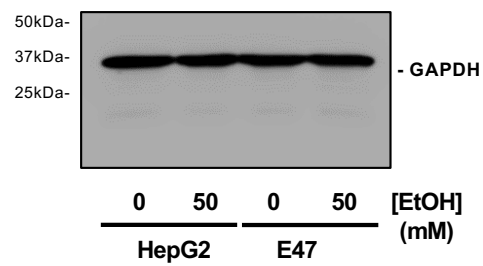

Supplement: Supplementary file 1 [file biology-11-01613-s001.zip › Supplemental Figures.pdf]
